# Destabilization of the Bacterial Interactome Identifies Nutrient Restriction-Induced Dysbiosis in Insect Guts

**DOI:** 10.1128/spectrum.01580-21

**Published:** 2022-01-05

**Authors:** Ramona Marasco, Marco Fusi, Matteo Callegari, Costanza Jucker, Francesca Mapelli, Sara Borin, Sara Savoldelli, Daniele Daffonchio, Elena Crotti

**Affiliations:** a Biological and Environmental Sciences and Engineering Division (BESE), Red Sea Research Center (RSRC), King Abdullah University of Science and Technology (KAUST), Thuwal, Saudi Arabia; b Department of Food, Environmental and Nutritional Sciences (DeFENS), University of Milan, Milan, Italy; University of Nebraska-Lincoln

**Keywords:** nutrient restriction, dysbiosis, bacterial microbiome, beta-diversity, dispersion, black soldier fly, interactome, co-occurrence network, gut, keystone species

## Abstract

Stress-associated dysbiosis of microbiome can have several configurations that, under an energy landscape conceptual framework, can change from one configuration to another due to different alternating selective forces. It has been proposed—according to the Anna Karenina Principle—that in stressed individuals the microbiome are more dispersed (i.e., with a higher within-beta diversity), evidencing the grade of dispersion as indicator of microbiome dysbiosis. We hypothesize that although dysbiosis leads to different microbial communities in terms of beta diversity, these are not necessarily differently dispersed (within-beta diversity), but they form disrupted networks that make them less resilient to stress. To test our hypothesis, we select nutrient restriction (NR) stress that impairs host fitness but does not introduce overt microbiome selectors, such as toxic compounds and pathogens. We fed the polyphagous black soldier fly, *Hermetia illucens*, with two NR diets and a control full-nutrient (FN) diet. NR diets were dysbiotic because they strongly affected insect growth and development, inducing significant microscale changes in physiochemical conditions of the gut compartments. NR diets established new configurations of the gut microbiome compared to FN-fed guts but with similar dispersion. However, these new configurations driven by the deterministic changes induced by NR diets were reflected in rarefied, less structured, and less connected bacterial interactomes. These results suggested that while the dispersion cannot be considered a consistent indicator of the unhealthy state of dysbiotic microbiomes, the capacity of the community members to maintain network connections and stability can be an indicator of the microbial dysbiotic conditions and their incapacity to sustain the holobiont resilience and host homeostasis.

**IMPORTANCE** Changes in diet play a role in reshaping the gut microbiome in animals, inducing dysbiotic configurations of the associated microbiome. Although studies have reported on the effects of specific nutrient contents on the diet, studies regarding the conditions altering the microbiome configurations and networking in response to diet changes are limited. Our results showed that nutrient poor diets determine dysbiotic states of the host with reduction of insect weight and size, and increase of the times for developmental stage. Moreover, the poor nutrient diets lead to changes in the compositional diversity and network interaction properties of the gut microbial communities. Our study adds a new component to the understanding of the ecological processes associated with dysbiosis, by disentangling consequences of diets on microbiome dysbiosis that is manifested with the disruption of microbiome networking properties rather than changes in microbiome dispersion and beta diversity.

## INTRODUCTION

In healthy animals, gut microbial communities promote host development, nutrition, growth, and homeostasis favored by compositional and functional diversity (1, 2). Polyphagous animals, which may experience periodic compositional changes in diet, have variable microbiome configurations that are all defined as normobiotic (3) if they support healthy conditions of the host (2–7). Considering the conceptual energy landscape, microbiome configurations can shift when selective forces are stronger than forces driving stability (1). Such alternative gut microbiome configurations can be considered neutral for host health when they continue to support resistance to perturbations and resilience (1, 2). However, normobiotic healthy microbiome configurations could be replaced by those associated with dysbiosis (1, 8) when the new microbiome configurations are not anymore able to support resistance to perturbation (2, 9–11), resulting in alterations of host health and fitness.

Diet is known to modify the gut microbiome (4, 6, 12–15), even in configurations that are associated with disease (1, 2, 15–18). The factors driving diet-induced changes in microbiome configurations in the absence of external negative effects of pathogens, parasites, or toxic compounds are not well understood. Many studies have assessed the effect of specific nutrients on diet, including fats, fibers, and vitamins (16, 17) also produced by gut bacteria. For instance, in *Drosophila* an acetic acid bacterium able to rescue the development of offspring reared on a thiamine-free diet (18) represents a potential dysbiosis-sensitive element of the *Drosophila* microbiome (i.e., when impaired, the insect host may lose a nutritional factor essential for growth). However, the conditions altering the microbiome composition and configuration in response to diet changes, particularly in polyphagous animals that rely on complex and variable diets, are elusive and difficult to be determined. Microbial diversity parameters, such as community dispersion, have been proposed as compendious indicators of dysbiosis (19). A weakness of compositional diversity parameters is that they overlook the interactions established by microbiome community members that are instead considered in interactome network assessments. We hypothesize that in animals equipped with complex microbiomes, including a large range of commensal microorganisms (20–22), the loss of resilience in diet-induced dysbiosis is mainly caused by the rearrangement of microbial communities and within-community interactions rather than modifications in community dispersion (19). We consider that alternative dysbiotic configurations that are induced by diet should have consistent dispersion because they are driven by a deterministic selection (e.g., diet components; (19)), but lower resilience because of weaker network interactions.

In this study we aimed to assess whether exposure to diet-related stressors determines (i) new configurations of the gut microbiome with consistent dispersion or stochastic changes with greater dispersion (19) and (ii) a stable or disrupted interactome network among members of the microbiome. To test our hypothesis, we used the saprophagous and generalist detritivore black soldier fly (BSF), *Hermetia illucens*, which is unconstrained in its nutritional requirements by specific diets (23–26). The BSF is a model organism in agricultural and biotechnological studies due to the capability of its polyphagous larvae to efficiently convert low-quality feed substrate (e.g., food waste, manure, and feces) into high-value biomass (24, 27). This capability is influenced by the presence and abundance of specific bacterial taxa within the gastrointestinal tract that are fundamental for bioconversion processes (28, 29). The nutritional versatility of BSFs results in a rich and diverse bacterial microbiome (30, 31), with widespread potential for complex bacterial networking (32). We compared the growth and development of BSFs under two different conditions of nutrient restriction (NR), namely, fresh fruit and vegetable NR diets (NRF and NRV, respectively), and a normal full-nutrient (FN) diet. We chose NR as a representative challenging condition because it does not introduce external negative effectors or selectors of the microbiome, but may alter the physicochemical conditions of the gut and exert adverse effects on insect growth and development (33, 34). We assessed the changes in bacterial diversity and interactome at different stages of the BSF life cycle (i.e., larvae, pupae, and adults) under the three diets (FN *versus* NRF *vs.* NRV) to link the observed changes in bacterial composition, structure, and networking with the fitness and growth performance of the host.

## RESULTS

### NR affects the development and growth performance of the insect.

**FIG 1:**
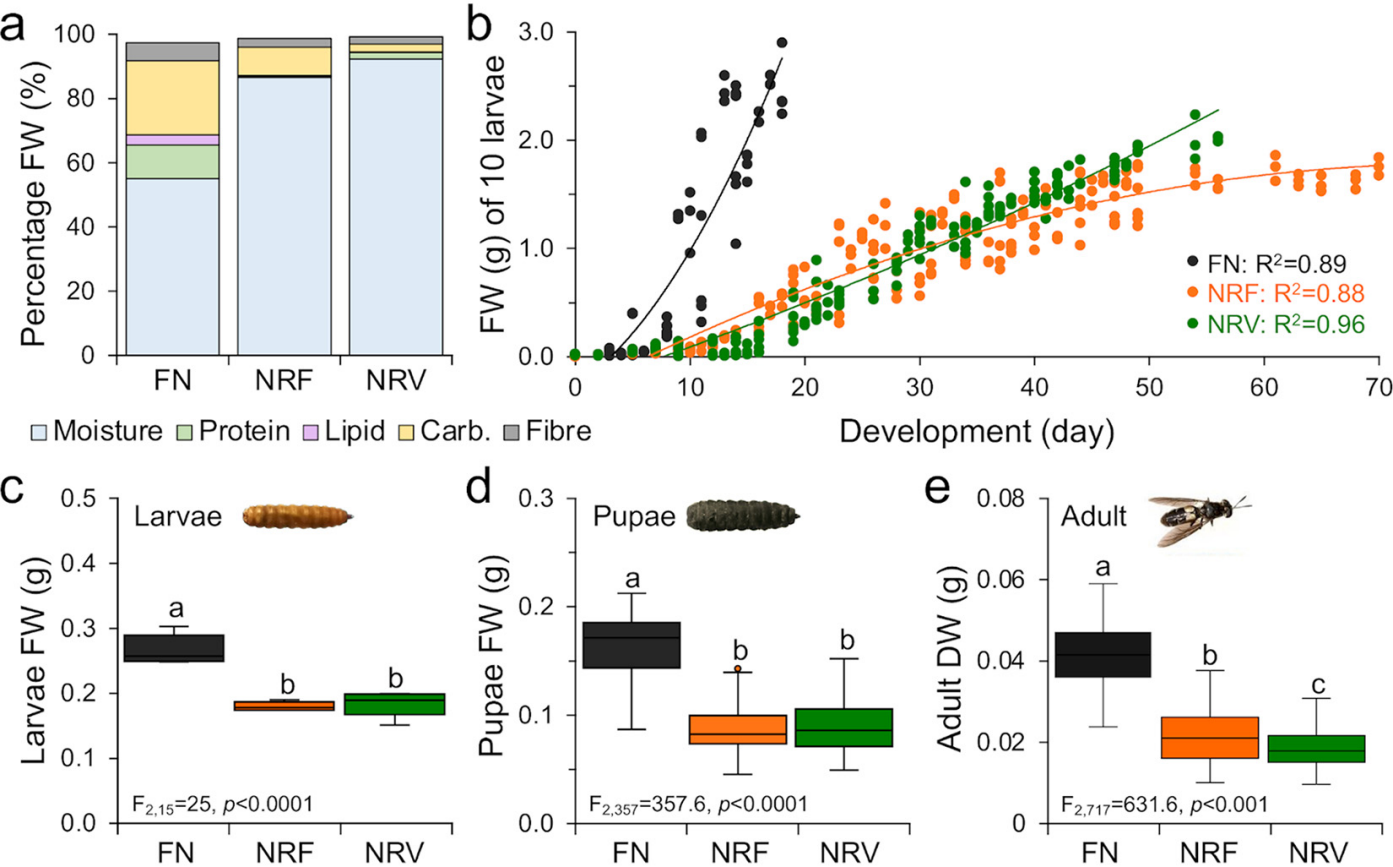
Influence of diet on BSF growth and development. (a) Chemical composition of the rearing substrates. FN, full nutrient; NRF, nutrient restriction fruit; NRV, nutrient restriction vegetable; Carb, carbohydrate. (b) Larval growth rate; the fresh weight of larvae (FW) fed with FN, NRF, and NRV diets are reported as a function of time (days of growth). The correlation coefficient (R^2^) of these two variables (FW and days) is reported for each diet in the graph; all correlations show significance probability, *P *< 0.0001. (c and d) FW of larvae and pupae and (e) dry weight of adults are presented as average ± standard deviation for the three diets (FN, NRF, and NRV). Weight is expressed in grams (g). Lowercase letters indicate the results of the Tukey’s multiple comparison tests among the diets (significance, *P *< 0.05).

Both of the two NR diets had lower nutrient content (i.e., proteins, lipids, carbohydrates, and fiber) than the control FN diet; however, they had different carbohydrate-to-protein ratios: NRF had more carbohydrates, while NRV had more proteins and moisture (Fig. 1a and Table S1 in the supplemental material). Both NR diets resulted in limited availability of food energy compared with FN (−77% and −88% kcal/g in NRF and NRV, respectively), with fewer calories per day (−79% and −92% kcal/day/larvae; Table 1). The energy limitations of NRF and NRV diets induced significantly longer larval–prepupal development times (ANOVA, F_2,15_ = 32.5, *P *< 0.0001; Table 1) and lower larval growth rates (Fig. 1b), with a final significant reduction in insect body size at each stage of the BSF life cycle compared to FN diet-fed individuals (ANOVA, weight, larvae: F_2,15_ = 47, *P *= 0.0002; pupae: F_2,357_ = 357, *P *< 0.0001; adult: F_2,717_ = 631.6, *P *< 0.0001; Fig. 1c–e; and length, pupae: F_2,177_ = 168, *P *< 0.0001; adult: F_2,357_ = 253, *P *< 0.0001; Table S2; e.g., Fig. S1). The growth performance parameters (i.e., weight) were negatively correlated with moisture and positively correlated with all other diet components (protein, lipid, carbohydrate, and fiber; Table S3). The waste reduction and bioconversion of experimental substrates by BSF larvae significantly differed between FN and NR diets (ANOVA, waste reduction index [WRI]: F_2,6_ = 2162, *P *< 0.0001; substrate reduction: F_2,6_ = 140, *P *< 0.0001; efficiency of conversion of ingested food [ECI]: F_2,6_ = 538.4, *P *< 0.0001; Table 1). For example, larvae more efficiently consumed FN substrates (highest WRI and ECI), with 2- and 5-fold lower substrate reduction compared with those fed with NRV and NRF diets, respectively (Table 1). Notably, NR diets had no effect on larval survival to the prepupal stage (ANOVA, F_2,6_ = 2.27, *P *= 0.18), but significantly less adult emergence (ANOVA, F_2,6_ = 87, *P *< 0.0001; NRF = −13% and NRV = −40%) and adult survival were observed (ANOVA, F_2,6_ = 66.5, *P *< 0.0001; NRF = −8% and NRV = −36%; Table 1).

**TABLE 1:** Substrate consumption and developmental performances of FN- and NR-fed BSF individuals^*a*^

Measurement	Analysis performed	Diet		
		FN	NRF	NRV
Diet energy (kcal/g)^*b*^		1.62	0.38 (−77%)	0.19 (−88%)
Consumption	Substrate reduction (%)	60.5 ± 4.0 (a)	86.3 ± 0.7 (b)	91.1 ± 0.9 (b)
	kcal/day/larva	0.111 ± 0.006 (a)	0.023 ± 0.0004 (b)	0.008 ± 0.0008 (c)
	ECI	0.29 ± 0.02 (a)	0.04 ± 0.001 (b)	0.08 ± 0.01 (c)
	Waste reduction index (WRI)	4.22 ± 0.11 (a)	1.04 ± 0.01 (b)	1.63 ± 0.02 (c)
Developmental stages	Larval development time^*c*^	16 ± 2 (a)	66 ± 19 (b)	54 ± 2 (b)
	Larval survival to prepupal stage^*d*^	94 ± 5 (a)	94 ± 1 (a)	92 ± 7 (a)
	Adult emergence^*e*^	92 ± 7 (a)	73 ± 2 (b)	47 ± 2 (c)
	Total survival^*f*^	86 ± 4 (a)	68 ± 2 (b)	44 ± 4 (c)

a BSF, black soldier fly; FN, full nutrient, NR, nutrient restriction; NRF, fruit NR; NRV, vegetable NR; ECI, efficiency of conversion of ingested food.

b Based on general Atwater factors from http://www.fao.org/3/y5022e/y5022e04.htm#fnB9.

c Time from egg hatching to 40% of prepupal stage (days) obtained from three replicates.

d To prepupal stage.

e From prepupal stage.

f Young larvae–adults. Values are reported as average ± standard deviation. Lowercase letters indicate results of Tukey’s multiple comparison tests among diets.

### Physicochemical conditions of guts in BSF larvae fed with FN and NR diets.

**FIG 2:**
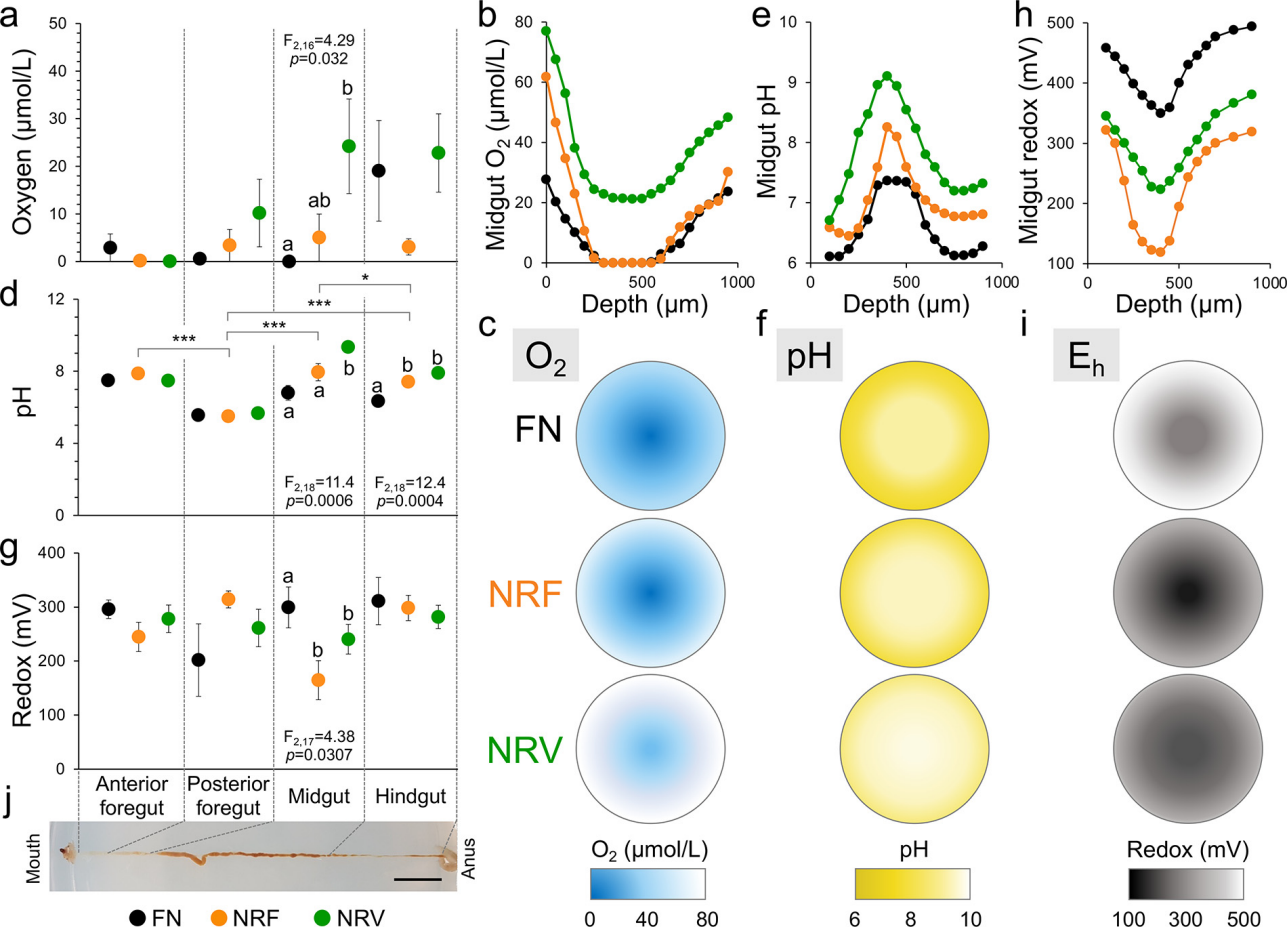
Physiological condition of BSF gut compartments. Microprofiles of (a–c) oxygen partial pressure (μm/L), (d–f) pH and (g–i) redox potential (mV) along the gut compartments of larvae reared on full nutrient (FN, black), nutrient restriction fruit (NRF, orange), and nutrient restriction vegetable (NRV, green) diets. (a, d and g) Values are given as average ± standard error (*n *= 7). (b, e and h) Representative radial profiles of oxygen partial pressure, pH, and redox potential, respectively, in the midgut of BSF fed on the three diets. Depth (μm) refers to the sensor tip position along the midgut. (c, f and i) Under the radial profiles, a schematic representation of oxygen (light blue spheres), pH (yellow spheres), and redox (gray spheres) gradients is reported, showing the effect of the three diets on the physiological status of the midgut. (j) Representative image of BSF gut. Scale bar 1 cm.

Considering the postfeeding conditions of BSF pupae (35) and the morphogenetic events affecting BSF adults (36), we determined the physicochemical conditions of oxygen partial pressure, pH, and redox potential of the larval gut only. Interestingly, among all the examined parts of the gut, significant changes in the examined parameters were only observed in the midgut (Fig. 2). Lacking the exoskeletal lining, due to its endodermal origin, the midgut is the primary site of digestion and nutrient absorption (28, 37). For instance, while oxygen concentration was close to zero in the gut lumen of FN-fed individuals, a higher variability was measured in the midgut of NR-fed larvae (ANOVA, F_2,16_ = 4.29, *P *= 0.032), with oxygen concentrations reaching up to 20 μmol/L in NRV-fed insects (Fig. 2). Although the increment in oxygen concentration in NR midguts was minimal, such changes can have detrimental effects on larval growth and molting (38, 39). We also detected changes in gut lumen pH, with diet having a significant effect in the distal portions of the gut (ANOVA, midgut: F_2,18_ = 11.41, *P *= 0.0006, and hindgut: F_2,18_ = 12.41, *P *= 0.0004; Fig. 2); we recorded a more alkaline pH in NR-fed larvae (up to 9.1 and 9.9 in NRF- and NRV-fed larvae, respectively) than in FN-fed larvae (for example, the midgut in Fig. 2). Such pH changes might influence nutrient availability by affecting the performance of the larval gut enzymes (such as proteases) (40). We also measured differences in redox potential in the larval midgut (ANOVA, F_2,17_ = 4.38, *P *= 0.03). We recorded significantly lower redox values in the midgut of NR-fed insects (mean, 165 and 240 mV in the NRF- and NRV-fed insects, respectively) than in the FN-fed insects (299 mV; Fig. 2), indicating possible changes in the microbiome and host metabolisms in this gut compartment (41, 42).

### Effect of NR on the bacterial community structure of BSF guts.

**FIG 3:**
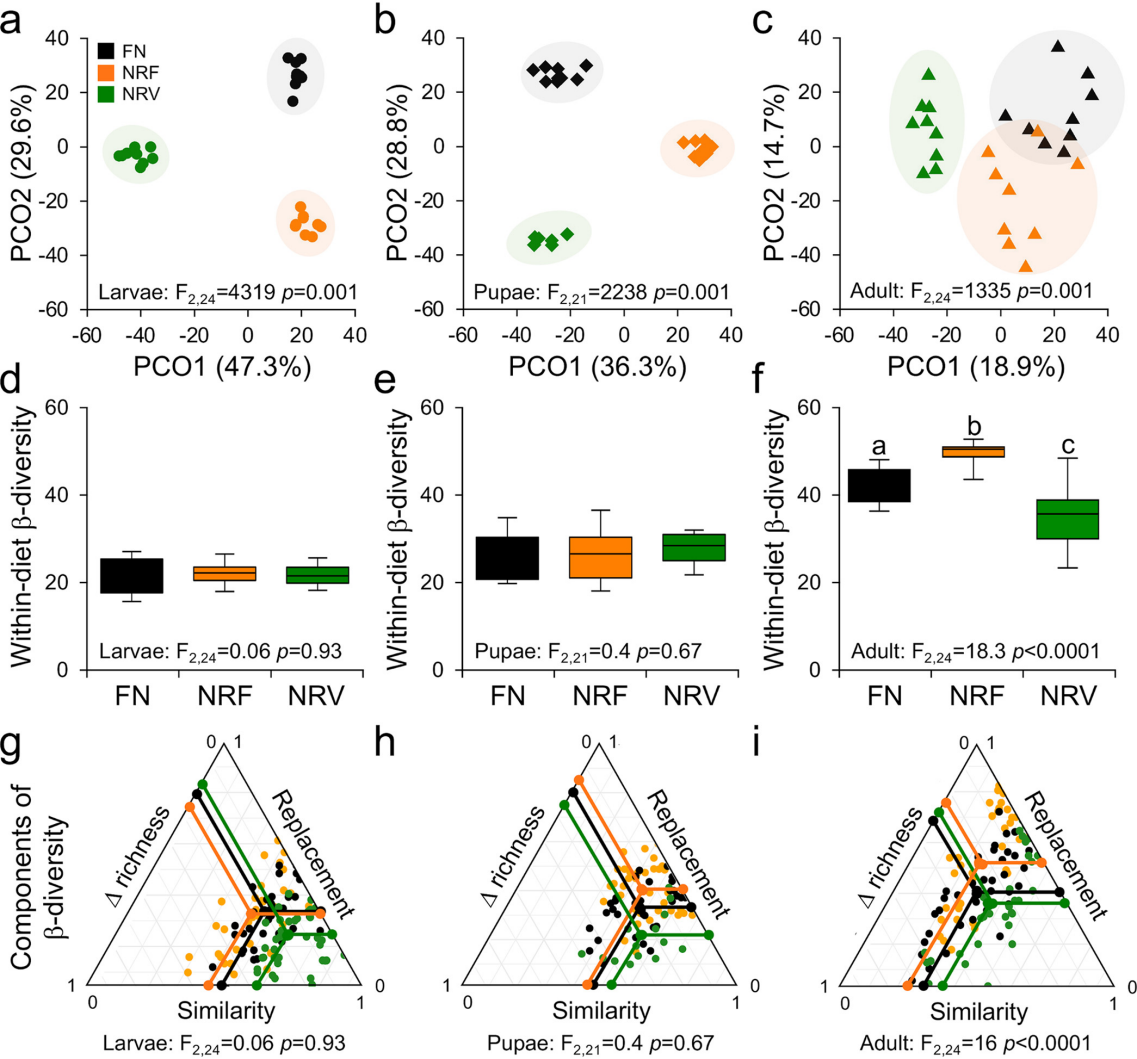
Bacterial diversity associated with BSF fed with different diets across the developmental life cycle. (a–c) Principal coordinates analysis (PCoA) based on the Bray–Curtis dissimilarity matrices of bacterial OTU tables. Each symbol corresponds to one sample of a given developmental stage (a: larvae, circle; b: pupae, diamond: c: adult, triangle), and their colors indicate the different diets (black, full nutrient [FN]; orange, nutrient restriction fruit [NRF]; green, nutrient restriction vegetable [NRV]). Results of multivariate analysis (GLM, general linear model) were also reported for each developmental stage. (d–f) At each stage, variations of within-beta diversity (sample dispersion) were measured as the distance of each sample from the centroid of each diet group. The distribution of within-beta diversity for each diet was visualized using boxplots (reported data: minimum, first quartile, median, third quartile, and maximum) in (d) larvae, (e) pupae, and (f) adults. Different lowercase letters above each boxplot denote a significant mean difference in dispersion based on the pairwise Tukey’s test at *P *< 0.05. ANOVA results were also reported. (g–i) Components of beta diversity (similarity, replacement, and richness difference). Triangular plots were used to visualize the relationships among the pairs of individuals for each diet in (g) larvae, (h) pupae, and (i) adults. Each point (FN, black; NRF, orange; NRV, green) represents a pair of samples within the diet. Its position is determined by a triplet of values from the similarity, replacement, and richness difference. In each triplet, the large central dots from which the lines start (black, orange, and green) are the centroid of the points; the lines represent the mean values of the similarity, replacement, and richness difference components.

The effects of NR on the growth and development of BSFs, as well as the variable physicochemical gut conditions, were also reflected in the structure and composition of gut bacterial communities. In each developmental stage, the bacterial communities associated with NR-fed guts formed distinct clusters (the 16S rRNA gene in Fig. 3a–c and 16S–23S rRNA internal transcribed spacers [ITS] in Fig. S2; statistical analysis in Tables S4), with diet explaining up to 36% of the total microbiome diversity observed (Table S5). Among the diet components, the higher concentration of moisture in NR diets, in addition to lower supply of carbohydrates and protein in NRV and NRF, respectively, significantly explained the differences in bacterial communities during BSF development (Table S6). The observed changes in bacterial communities (Bray–Curtis similarity) also significantly correlated with the deterioration of host health (i.e., weight loss; Fig. S3), indicating an NR-driven dysbiosis starting from the initial phase of insect development. Differences in richness (i.e., the number of operational taxonomic units, OTUs; Table S7) between FN- and NR-fed BSFs affected bacterial community similarity at all stages of the BSF life cycle (richness decay in Fig. S4) but did not influence community dispersion (i.e., within-beta diversity; Fig. S5). The stressful conditions of NR diets shifted the gut bacterial community configurations from one stable state to another (19). During the juvenile stages (larvae and pupae), FN-fed individuals had relatively stable bacterial communities that formed tight clusters in the ordination space (Fig. 3a and b). On the other hand, NR diets determined distinct clusters with similar dispersion to FN (location effects; Fig. 3d and e); according to Zaneveld et al. (19), such new microbiome configurations driven by the diet type are defined as deterministic, because they had similar dispersion (within-beta diversity) to the nonperturbed condition (the FN diet). On the contrary, in the adults where the morphogenetic events affect/degenerate the internal tissues of insects (36), NR diets significantly affected the sample-to-sample variability (PERMDISP: F_2,24_ = 18.3, *P *< 0.0001), with destabilization of the bacterial communities and further increment in heterogeneous selection, either increasing (NRF) or decreasing (NRV) microbiome dispersion (Fig. 3c and f), indicating the effect of stochastic processes (19) that cannot be predicted precisely.

In larvae, pupae, and adults, the within-diet beta diversity was primarily driven by OTU replacement, defined as substitution of species in one treatment by different species in another treatment (43, 44), with an average relative contribution of 60%; richness differences, defined as gain and loss of species between treatments (43, 44), followed with an average contribution of 40% (Fig. 3g–i and Table S8). This indicates that the different diets drive a significant environmental filtering process induced by the alteration of gut physicochemical conditions (Fig. 2) and the change of bacterial communities’ diversity, as well as by their reciprocal influence. This pattern was consistent across all BSF developmental stages for all three diets, but with different magnitudes (larvae: F_2,105_ = 14.5, *P *= 0.001; pupae: F_2,84_ = 8.9, *P *= 0.001; adult: F_2,105_ = 6.6, *P *= 0.001; Fig. 3g–i), evidencing an overall higher rate of replacement for the NRF diet (up to 74%) than for the FN and NRV diets (up to 63% and 58%, respectively; Table S8).

### NR diets alter the bacterial community composition of BSF gut.

**FIG 4:**
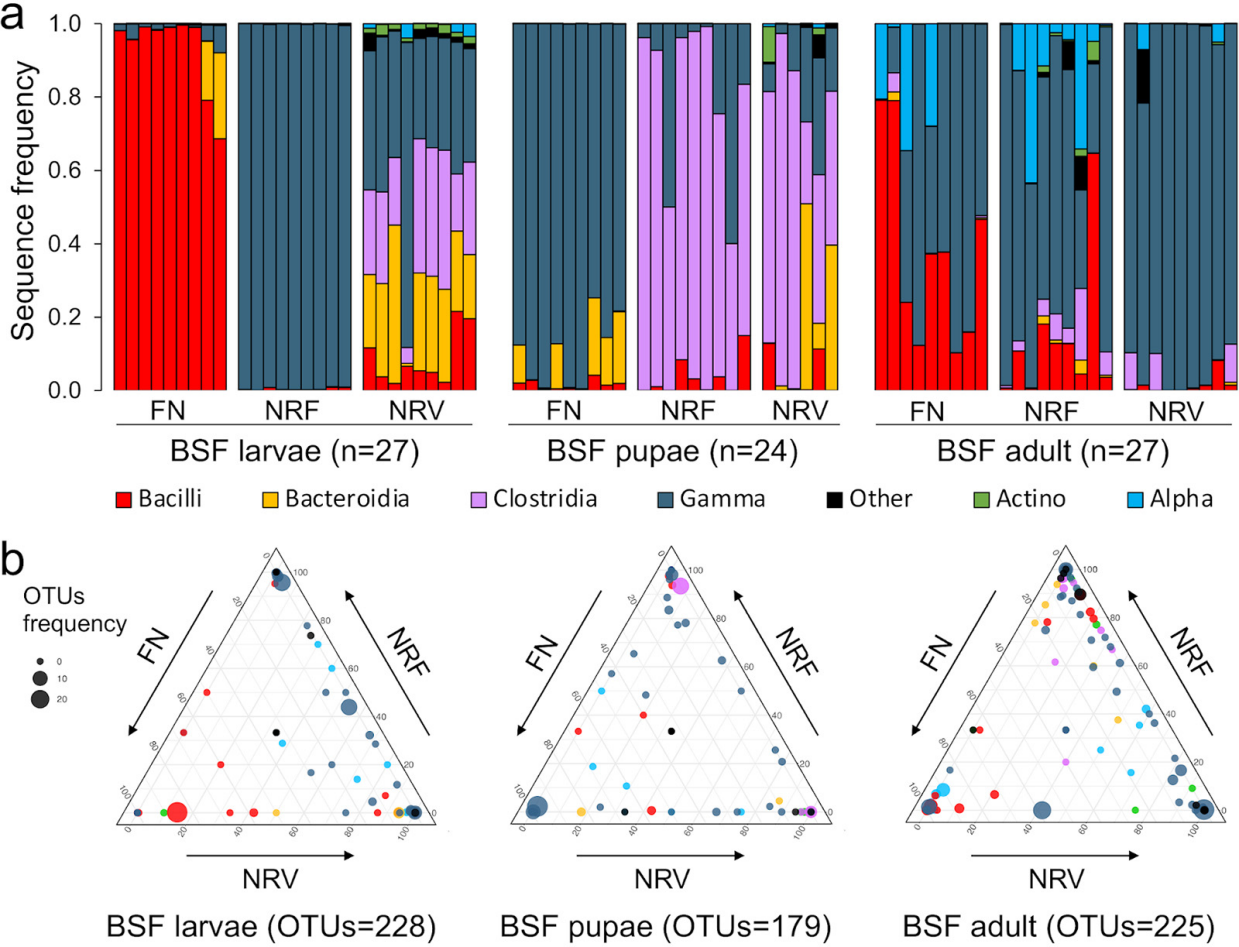
Bacterial community composition according to BSF developmental stage and diet conditions. (a) Relative abundance of the bacterial class across BSF developmental stages under the three diets (FN, full nutrient; NRF, nutrient restriction fruit; NRV, nutrient restriction vegetable). Relative abundance is expressed as a percentage of sequence frequency. (b) Ternary plot of all OTUs. Each ternary plot graphically depicts the relative distribution of OTUs (indicated by dots) across the three diets, namely, FN, NRF, and NRV. In each triangle, the three axes indicate the three different diets; the position of the dots within the triangle area is determined by the contribution of the three diets to the total relative abundance. The relative contributes of each diet (range, from 0% to 100%) are extrapolated by interpolating parallel lines to the three axes passing from a given dot; the sum of the three values is 100%. The size of the dots indicates the OTUs frequency (i.e., number of samples having a given OTU), while the color indicates their taxonomic affiliation at the class level. Relative abundance of all of the bacterial OTUs across the samples is reported in Fig. S7.

As the NR diet-induced dysbiosis progressed, the composition and structure of the BSF gut bacterial communities changed (relative distribution in Fig. 4a and Fig. S6a and S7), establishing diet-specific bacterial sub-communities during BSF development (spheres in the ternary corners; Fig. 4b). In the juvenile stages (larvae and pupae), the main difference between the healthy (FN) and dysbiotic microbial communities (NRF and NRV) was a drastic depletion in the main components of the bacterial community (*Bacilli* and *Gammaproteobacteria* in larvae and pupae, respectively) compared with other community members. For instance, in BSF larvae, the class *Bacilli*—dominant in FN-fed individuals (93%, 9%, and 1% in FN, NRV, and NRF, respectively)—was replaced with a combination of *Gammaproteobacteria* (*Enterobacteriaceae*), *Bacteroidia* (*Porphyromonadaceae*), and *Clostridia* (*Lachnospiraceae*, *Ruminococcaceae*, and Family XI) in NRF- and NRV-fed individuals (Fig. 4a, Fig. S6 and S8, Data Set S1). In the pupal stage, *Clostridia* bloomed in NR-fed individuals, suppressing the dominant *Gammaproteobacteria* (*Enterobacteriaceae*) typical of FN-fed pupae (Fig. 4a). In adults, *Gammaproteobacteria* prevailed in all three diets (52%, 64%, and 92% in FN, NRF, and NRV, respectively), along with the presence of *Bacilli* in FN-fed individuals (34%, 14%, and 2% in FN, NRF, and NRV, respectively) and *Alphaproteobacteria* (*Acetobacteriales*, 17.7%, 6.4%, and < 1% in FN, NRF, and NRV, respectively), *Clostridia* (< 1%, 5%, and 4% in FN, NRF, and NRV, respectively), and *Alphaproteobacteria* (*Sphingomonadales*, < 0.01%, 6.5%, and 1.3% in FN, NRF, and NRV, respectively) in NR-fed individuals (Fig. 4a). Although changes in relative abundance can be interpreted as changes of specific OTUs, they could be the result of decreases/increases in other community members rather than, or in addition to, changes in absolute abundance of specific bacterial OTUs. Notably, the three diet substrates (i.e., flours, fresh fruits, and fresh vegetables in FN, NRF, NRV, respectively) were all dominated by members of *Alphaproteobacteria* (range, 35%–77% of relative abundance), followed by *Actinobacteria* (17%–41%), *Gammaproteobacteria* (3%–14%), *Bacilli* (3%–26%), and *Bacteroidia* (2%–15%; Fig. S6b; Data Set S1). Although the substrate was assumed to be an important source of bacteria (29, 43), our results showed that the bacterial composition of diets was not mirrored within the bacterial communities associated with juvenile and adult stages. Moreover, the absence of a prevalent generalist community in favor of diet-specific bacterial sub-communities (Fig. 4b) and the strong correlation between bacterial community members and diet components and growth (Table 2; Result S1) confirmed that the bacteria in the BSF gut were strongly selected by both NR and the developmental stages.

**TABLE 2:** Correlation analysis between the main bacterial classes detected in larvae, pupae and adults and the different components of the three diets (full nutrient, nutrient restriction fruit and nutrient restriction vegetable) and growth performance of individuals (weight)^*a*^

Stage	Class	Moisture	Protein	Lipid	Carb.^*b*^	Fiber	Others	Wt^*c*^
Larvae	*Actinobacteria*	0.53**	–0.29	–0.39*	–0.64***	–0.50**	–0.62**	–0.33
	*Alphaproteobacteria*	0.33	–0.18	–0.25	–0.40*	–0.31	–0.38*	–0.25
	*Bacilli*	–0.96***	0.98***	0.98***	0.91***	0.97***	0.92***	0.94***
	*Bacteroidia*	0.37	–0.15	–0.24	–0.48*	–0.35	–0.46*	–0.20
	*Clostridia*	0.55**	–0.32	–0.42*	–0.66***	–0.53**	–0.63***	–0.40*
	*Erysipelotrichia*	0.36*	–0.21	–0.28	–0.44*	–0.35*	–0.42*	–0.21
	*Gammaproteobacteria*	0.68***	–0.85***	–0.79***	–0.55**	–0.70***	–0.58**	–0.76***
	*Negativicutes*	0.33	–0.19	–0.25	–0.39*	–0.31	–0.38*	–0.33
	*Oxyphotobacteria*	0.28	–0.40*	–0.35	–0.20	–0.29	–0.22	–0.33
Pupae	*Actinobacteria*	0.25	–0.14	–0.18	–0.30	–0.23	–0.29	–0.31
	*Alphaproteobacteria*	0.25	–0.12	–0.17	–0.32	–0.24	–0.31	–0.38
	*Bacilli*	0.25	–0.24	–0.24	–0.25	–0.25	–0.25	–0.32
	*Bacteroidia*	–0.01	0.16	0.11	–0.05	0.05	–0.05	–0.28
	*Clostridia*	0.83***	–0.89***	–0.87***	–0.77***	–0.84***	–0.78***	–0.72***
	Deltaproteobacteria	0.13	–0.19	–0.17	–0.01	–0.14	–0.10	–0.22
	*Erysipelotrichia*	0.27	–0.15	–0.20	–0.33	–0.26	–0.32	–0.33
	*Gammaproteobacteria*	0.93***	–0.92***	–0.93***	–0.90***	–0.93***	–0.91***	0.75***
	*Negativicutes*	0.29	–0.16	–0.22	–0.35	–0.27	–0.34	–0.21
	*Oxyphotobacteria*	0.31	–0.24	–0.27	–0.35	–0.30	–0.34	–0.20
	*Saccharimonadia*	–0.27	0.27	0.27	0.26	0.27	0.26	0.05
Adult	*Actinobacteria*	0.22	–0.35	–0.30	–0.14	–0.24	–0.16	–0.39*
	*Alphaproteobacteria*	–0.24	0.16	0.19	0.28	0.23	0.27	0.13
	*Bacilli*	–0.61***	0.54**	0.58**	0.63***	0.61***	0.63***	0.52**
	*Bacteroidia*	0.05	–0.13	–0.05	0.01	–0.05	0.01	–0.11
	*Clostridia*	0.33	–0.37	–0.36	–0.30	–0.34	–0.30	–0.38*
	Deltaproteobacteria	0.10	–0.17	–0.15	–0.01	–0.11	0.05	–0.23
	*Erysipelotrichia*	–0.36	–0.35	–0.36	0.35	–0.36	0.35	0.39*
	*Gammaproteobacteria*	0.49**	–0.37	–0.42*	–0.54**	–0.48*	–0.53**	–0.38*
	*Negativicutes*	0.15	–0.25	–0.21	–0.01	–0.16	–0.01	–0.32
	*Oxyphotobacteria*	0.21	–0.19	–0.20	–0.22	–0.21	–0.22	–0.19
	*Saccharimonadia*	0.17	–0.10	–0.13	–0.21	–0.16	–0.20	–0.13
	*Verrucomicrobiae*	0.10	–0.17	–0.15	–0.05	–0.11	–0.05	–0.23

a Values indicated the correlation coefficient obtained by Pearson, Kendall and Spearman correlation tests and asterisks their statistical significance. *, *P *< 0.05; **, *P *< 0.01; ***, *P*< 0.001.

b Carb., carbohydrate.

c Refer to the weight reported in Fig. 1c–e for larvae, pupae, and adults reared under different diets.

### NR diets decrease network stability in the microbial community of BSF guts.

**FIG 5:**
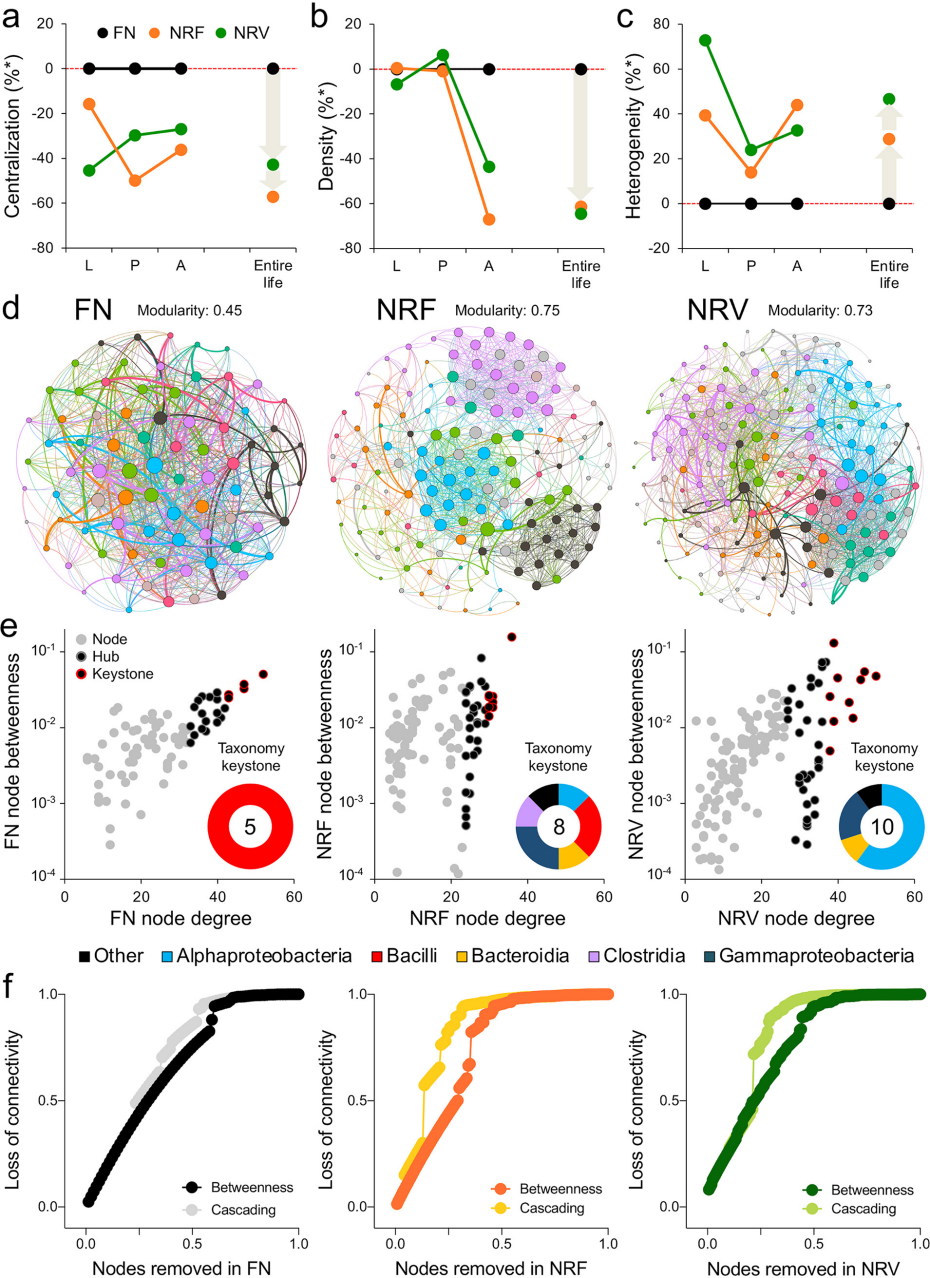
Interactomes of the bacterial communities associated with BSF individuals fed on different diets. (a–c) Topological indices (centralization, density, and heterogeneity) of the co-occurrence networks calculated for each developmental stage (L, larvae; P, pupae; A, adults) and the entire life span of BSF based on their feeding conditions (black, full nutrient [FN]; orange, nutrient restriction fruit [NRF]; green, nutrient restriction vegetable). Values are expressed as percentages compared with FN conditions (normobiotic). (d) Visualization of the bacterial co-occurrence networks of insects fed with FN, NRF, and NRV diets, considering the entire life span of BSF. Each node represents different bacteria (OTUs), and each edge represents significant co-occurrence relationships. Node size indicates the abundance of each OTU, whereas the colors indicate different modules; colors are assigned randomly to each module by the Gephi software and do not indicate differences in taxonomy. (e) For each diet, the nodes identified as hubs (black dots with gray border) are those that are more central based on their degrees, i.e., degree of connection >75%; those with the highest level of degree of connection and betweenness centrality are classified as keystones (black dots with red border). Taxonomic affiliation of keystone nodes is also reported in pie charts. (f) Robustness of the bacterial networks in the whole life of insects fed with the three different diets. We stepwise removed the nodes and recorded the loss of connections (edges) in the network. The removal of all the nodes (from 0% to 100%) from each network is displayed, after which the loss of connectivity was measured as remaining betweenness and cascading (i.e., betweenness recalculated after each removal).

To assess the biotic interactions among members of normobiotic (nonstressed FN) and dysbiotic (stressed NRs) bacterial communities in BSF guts, we performed co-occurrence network analyses. Despite the observed variability in the number of nodes and interactions among the developmental stages and feeding diets (developmental stages in Table S9 and entire life in Table 3), we observed a consistent increase of nodes and OTU–OTU interactions in the NR-fed individuals compared to the FN-fed individuals, possibly due to their similar environmental preference and/or the high resistance of gut microorganisms (commensals) to physiological gut modifications induced by the diets (Fig. 2). The NR gut bacterial interactomes were consistently more rarefied (lower centralization), less structured, and less connected (lower heterogeneity and density) than the FN gut bacterial interactomes across all developmental stages (including the entire life span of the insect; Fig. 5a–d, Table 3, and Table S9). Due to these consistent disaggregation patterns, the co-occurrence networks obtained from the analysis during the entire life span of the insects were considered to determine the main taxa involved in the structuring of the interactomes under the three diets. As expected, the composition of the FN and NR interactome nodes differed, partially reflecting the bacterial taxonomic diversity described for the entire community (Fig. S9). Regardless of their abundance, the most influential OTUs within the network were those with higher connections (i.e., hubs and keystones). In particular, these OTUs—involved in community assembly, stability, and functionality (44)—varied according to diet administration (Fig. 5e and Fig. S9); for example, the keystone OTUs of FN-fed individuals were all affiliated with *Bacilli* (i.e., *Leuconostoc*, *Lactobacillus*, *Bacillus*, and *Weissella* genera), while those of NR-fed individuals were more variable, encompassing members of *Clostridia* (Family XI) and *Gammaproteobacteria* (Acinetobacter, *Beggiatoaceae*, and *Burkholderiaceae*), as well as members of less abundant groups, such as *Alphaproteobacteria* (*Devosia*, *Ochrobactrum*, *Kaistia*, and *Sphingomonas*), *Bacteroidia* (*Dysgonomonas* and *Myroides*) and others (*Leucobacter* and *Flaviflexus* genera of *Actinobacteria*). A stepwise removal of nodes and measurement of the loss of connections (edges) in the networks revealed that in the NR diets up to 90% and 89% of stability (i.e., robustness) was lost after the removal of 30% of the nodes in NRF and NRV, respectively (Figure 5f); the same node removal approach caused a loss of only 58% of connectivity in the FN network. This result indicated that in NR diets the networks were highly disconnected, leading to a substantial disaggregation of the network.

**TABLE 3:** Topological indices describing the co-occurrence networks of the entire life span of FN-, NRF-, and NRV-fed BSF individuals^*a*^

Topological indices	Diet		
	FN	NRF	NRV
No. of nodes	81	140	166
No. of interactions	1011	1129	1505
Positive	622 (62%)	1004 (89%)	1107 (74%)
Negative	389 (38%)	125 (11%)	398 (26%)
Mean of degree ± SD	25 ± 11 a	16 ± 9 b	18 ± 12 b
Cluster coefficient^*b*^	0.58	0.67 (+16%)	0.59 (+2%)
Centralization^*b*^	0.35	0.15 (−58%)	0.2 (−44%)
Avg path length^*b*^	1.77	2.61 (+47%)	2.46 (+39%)
Avg neighbors^*b*^	24.96	16.13 (−35%)	18.13 (−27%)
Density^*b*^	0.31	0.12 (−63%)	0.11 (−65%)
Heterogeneity^*b*^	0.45	0.58 (+30%)	0.66 (+47%)

a BSF, black soldier fly; FN, full nutrient; NRF, nutrient restriction fruit; NRV, nutrient restriction vegetable.

b Percentage of increment (+) and decrement (−) in NRF and NRV compared to FN are shown in brackets.

## DISCUSSION

The significance of dysbiosis and its importance in affecting the growth, health, and well-being of animals is widely debated and based on the hypothesis of a causal relationship between the microbiome configuration(s) in healthy individuals and their positive performance. However, such a causal relationship is hard to demonstrate due to the circularity of the following question (7): What comes first—poor host performance and unhealthy state or the associated microbiome configuration? In other words, is dysbiosis a cause or a consequence of poor health? In our study, we faced the same issue confounding most other studies on dysbiotic microbiomes. We imposed BSF to stressful diet conditions (i.e., NR) and then compared these conditions with those of BSF fed on an FN diet that supports good growth and development. We chose NR as a stressor to induce dysbiosis because this restriction strongly perturbs the host and its microbiome (45) without introducing external direct selectors of microbes, such as toxic chemical compounds or virulence factors produced by microbial pathogens or parasites. Our assumption is that by putting the host/microbiome holobiont under stress with NR diets, we could modify the gut microbiome (46, 47) to “detrimental configurations,” which would weaken host resilience (2, 9), a requisite for defining the change in microbiome configuration as dysbiotic (19).

In our study, we used two unrelated NR diets that result in nutrient and caloric restriction stresses to the BSF. The FN diet was optimal in sustaining insect growth and development throughout the life cycle; this allowed us to obtain heavier insects in a shorter time, due to the higher carbohydrate, lipid, and protein contents in the FN diet compared to the NR diets (34, 37, 48, 49). The good growth performance of the host suggests that the gut microbiome configuration under the FN diet is normobiotic (3), leading to the circular definition considered above; therefore, the microbiome configurations identified under the NR conditions are dysbiotic (as indicated by poor BSF growth performance and altered gut physicochemical conditions in Fig. 1 and 2, respectively).

The interpretation issue of the role and competence of a microbiome configuration in dysbiosis is due to the lack of a univocal rather than comparative definition of dysbiosis (15). However, the general variability in selection conditions, including host genotype, variable diets, host immune system, and different environmental conditions under which the host lives (45, 50–54), suggests that there can be multiple normobiotic and dysbiotic microbiome configurations, with a full causative effect on host performance and health (11, 19). Experiments conducted using variable diets showed how the nutritional complexity of the substrate and high bioburden levels favor the overall diversity of the bacterial communities associated with BSF individuals (29, 43, 46). Although the substrate is assumed to be an important source of bacteria, the diets we have used here to feed BSF were prepared with fresh ingredients rather than wastes, which generally have much higher microbial titers than fresh non-fermented materials. Thus, we consider that in our experiments, such fresh ingredients may not have contributed to define the observed microbial diversity in the BSF gut because of their bioburden, but rather, because of their compositional and caloric features.

The extreme variability observed for the BSF microbiome and its limited similarity with the rearing substrates (29, 43, 55, 56) indicate that further (abiotic and biotic) factors than the diet and their interdependences have an important role in the assembly of the BSF gut microbiome (29, 43, 54, 57). For instance, the gut immune system acts as a barrier preventing random modification of the bacterial communities under changing conditions. A recent study showed that BSF larvae can produce a wide spectrum of antimicrobial peptides (AMPs), many of which are expressed in response to changing nutritional conditions or high bacterial loads (58). The authors hypothesized that this expanded spectrum and diet-dependent expression of AMPs is essential for BSF larvae’s adaption to nutritionally unpredictable substrates that are often highly contaminated with potential pathogens. Under the NR conditions that we have used in our experiments, we cannot exclude a disruption of the AMP response of the BSF that may have further worsened the dysbiotic conditions of the gut microbiome. This aspect could be explored in further studies that investigate the relationship between NR and gut dysbiosis in BSFs.

The above-mentioned factors associated with the complexity and variability in the BSF gut microbiome assembly can explain the differences among the experimental results obtained in different laboratories (30, 43). In some cases, the microbiota of the BSF was dominated by either Bacteroidetes, Proteobacteria, or Firmicutes (*Bacilli* and *Clostridia*), along with a variable amount of Actinobacteria, Fusobacteria, and unclassified bacteria (29, 46, 55, 56, 59–61). Experiments performed with substrates similar to our FN diet (i.e., cooked rice and Gainesville diet [46, 60]) and food waste (61) showed *Enterococcus* spp. (Firmicutes, *Bacilli*) to be one of the main components of the bacterial community (range, 20%–74% of relative abundance [29, 57, 59]), suggesting that it could be a critical player in the digestive process; *Enterococcus* strains secrete a variety of proteases and pectinases and are also involved in the degradation of indigestible carbohydrates in cereal and straw-related substrates (61). In addition, regardless of substrate used, a group of bacteria consistently present in BSF gut (i.e., core bacterial microbiome) was identified, possibly because they support a dietary flexibility and availability of antimicrobial peptides that represent a benefit for the insect in different environments (43, 45, 57, 59). In particular, *Providencia*, along with *Actinomyces* (Actinobacteria), *Dysgonomonas* (Bacteroidetes), and *Enterococcus* (Firmicutes), were reported as recurrent taxa that occur in BSF reared on different substrates (29, 56, 57). In our work, while bacterial OTUs belonging to *Providencia* and *Enterococcus* were detected across the entire life cycle of BSF reared on the three diets (overall mean, 14%), *Actinomyces* and *Dysgonomonas* OTUs were mainly found in individuals fed with NRV diet and rarely in individuals fed with FN (Data Set S1); these OTUs were not detected in diet substrates (Data Set S1) and were generally found in the substrate after its contact with larvae (62).

The multiple configurations of normobiotic and dysbiotic microbiomes can be subjected to changes driven by different conditions that favor the passage from one configuration to another due to changing selective forces (1, 7, 19). In this context, it has been proposed under the frame of the Anna Karenina Principle (AKP) (63) that stressful conditions and the related microbial configurations represent stochastic perturbations that can induce microbiome destabilization, thereby resulting in more dispersed microbial communities in stressed individuals (19). In NR-fed BSF juveniles (larvae and pupae), rather than observing such increments in dispersion (within-beta diversity; Fig. 3d and f), we observed alternative stable states of the microbiomes with similar dispersion driven by diet (i.e., distinct clusters in the ordination space: location effects) (19). Contrastingly, adult microbiomes were altered in unpredictable ways (i.e., with different dispersions). However, adults have a particular gut system that undergoes morphogenetic events, which in turn affect the internal tissues; this may pose some limitations on gut functionality (36). The location effect—driven by diet (deterministic change) rather than by the increment in dispersion (high intrinsic variability led by stochastic change) in NR-fed juvenile microbiome configurations—can be explained by the robustness of the immune system of the BSF as discussed above. Because the two NR diets strongly affected BSF growth and development and selected different microbiome configurations without affecting the range of dispersion, we conclude that within-beta diversity cannot be considered a consistent indicator of the unhealthy state of the BSF under NR diets. The results point out that under NR stress, BSF juvenile guts do not follow the AKP effects discussed by Zaneveld et al. (19) but establish new stable and alternative “stressed” bacterial community configurations driven by deterministic changes induced by NR diets.

According with our initial hypothesis, NR diets did not affect BSF gut bacterial community dispersion but did affect the bacterial interactome and, in particular, the centralization parameters of the network and its stability. A denser, more structured, more connected and more stable bacterial interactome was selected by the FN diet, a condition associated with efficiently performing microbiomes (64), including the counteraction of possible pathogens or opportunistic microorganisms (65, 66) and promotion of gut homeostasis (67). In contrast, networking of NR-fed individuals resulted in less structured configurations, with separate loosely interconnected modules—a condition more typical during stress (68, 69) that can negatively impact both the host and the function of the microbiome. In FN-fed individuals, *Bacilli* belonging to the genera *Leuconostoc*, *Lactobacillus*, *Weissella*, and *Bacillus* were denoted as the keystone species of the interactome. The first three of these genera are lactic acid bacteria and are known as favorable microorganisms due to their ability to stimulate host gastrointestinal development and digestive function (carbohydrate, peptide, and lipid metabolism), immune response, and improved disease resistance (56, 70, 71). Instead, in both the NR interactomes, *Bacteroidia*, *Gammaproteobacteria*, *Alphaproteobacteria*, and *Clostridia*, along with less abundant groups, were the main keystone taxa. These taxa have been previously found as dominant members in BSFs fed with food waste and poultry manure (54) and have also been proposed as potential microbial signatures of diseases (61).

Recent studies have tested the probiotics potential of some bacteria for the BSF, including taxa (such as *Bacillus* and *Lactobacillus*) that we have identified as keystone species in the network interactome of normobiotic individuals. Administration to the BSF of *Bacilli* isolated from the gut of healthy BSF larvae, such as Bacillus subtilis and B. licheniformis, has reportedly shortened the development period of BSFs from larval to adult stage and has significantly increased their growth when reared on poultry manure (72) and NR (73) diets, respectively. Similarly, the addition of Lactobacillus buchneri on soybean curd residue substrate increased the rearing performance of BSF larvae, also enhancing their nutritional value (71).

## CONCLUSIONS

The present study shows how dysbiosis induced by diet is primarily involved in shaping bacterial community composition of the BSF gut, but does not affect the community dispersion. NR determines changes of the gut bacterial community to new non-dispersed configurations that are dysbiotic because they do not support the host resilience. However, the members of these communities remodulate their interactions into rather disintegrated networks with low connections and stability, associated with decreased holobiont resilience and host homeostasis. We conclude that it is important to understand the variation and network properties of the microbiome associated with decreased host performance to confirm a microbiome dysbiotic condition and to assess the nature of the effect of unbalanced diets over microbial community configurations and host performance and resilience.

## MATERIALS AND METHODS

### Diet composition and insect growth.

BSF specimens were reared at the entomological facilities of the University of Milan using the methods described by Jucker et al. (34). Specimens were reared on three different diets: an FN diet, comprising 50% wheat germ, 30% alfalfa, and 20% corn flour, to which an equal volume of water was added; an NRF diet, comprising fresh apples, pears, and oranges (33.3% each); and an NRV diet, comprising fresh green beans, cabbage, and lettuce (33% each). The chemical composition of the three diets was further calculated in term of moisture, proteins, lipids, carbohydrates, fibers, and all remining components not previously listed (others). In the case of FN diet, the chemical composition of each ingredient was obtained from the provider Laboratorio Dottori Piccioni s.r.l. (Gessate, Milan) and used to calculate the overall chemical composition of the diet based on the percentage of each ingredient used; in the case of NRF and NRV diets, the chemical composition of each fruit/vegetable type was retrieved from https://www.alimentinutrizione.it/tabelle-nutrizionali/ricerca-per-categoria and used to calculate the overall chemical composition of the NR diets based on the percentage of each fruit/vegetable component. The two NR diets were selected because they had (i) lower nutrient contents (proteins, lipids, carbohydrates, and fiber) than those supplied by the control FN diet and (ii) different carbohydrate-to-protein ratios (an important diet parameter for BSF development (72); Table S1). In addition, NR diets were prepared using different ingredients from the FN diet (i.e., fresh fruits and vegetables) to exert a different selective pressure (45, 73) and mimic the variability of the natural diets to which the BSF could be exposed (e.g., horticulture waste) (34, 73, 74).

From the stock culture of a laboratory fly, the eggs of *H. illucens* were collected on cardboard strips for oviposition and then transferred into plastic containers (10.5 × 5 cm), with metal mesh to allow air exchange, containing the three diets (FN, NRF, and NRV). These containers were kept in a climate chamber under controlled conditions (temperature: 25°C ± 0.5°C; relative humidity: 60% ± 5%; photoperiod: 12:12 light:dark). The larvae (*n *= 400 per diet) were provided *ad libitum* access to the three diets. Emerging pupae were transferred into three different cages according to the feeding diet and without additional food sources (FN, NRF, and NRV) until the eclosion of adults. The following data were recorded: larval (measured after egg eclosion, when 40% of larvae reached the pupal stage), pupal, and adult weight (Sartorius CP64 analytical balance, Germany); pupal and adult length (mm); and the survival of individuals in each developmental stage. Fresh weights were determined for larvae and pupae, whereas dry weight was determined for adults after desiccation at 105°C for 48 h by using an analytical balance (Sartorius CP64, Germany). The efficiency of conversion of ingested food (ECI), the waste reduction index (WRI), and substrate reduction (SR) were calculated from the total amount of food added (W), the food remaining at the end of the experiment (R) and the final biomass of larvae and pupae (B) as follows: ECI = B/(W − R); WRI = (W − R/W) × days × 100; SR = W − R/W × 100. For larvae, the growth rate was also calculated and expressed as an average fresh weight (g) per day of growth. Correlation (Pearson, Kendall, and Spearman, *P *< 0.05) between growth performance (i.e., weight) and diet components was assessed using R software.

In addition to insect growth performance analysis, physicochemical conditions (oxygen partial pressure, pH, and redox potential) were also measured using the methods described in Method S1. For these measurements, only fourth instar larvae were used because gut functionality is not affected by a noncontinuous feeding behavior or by the remodeling processes that are associated with the subsequent developmental stages (i.e., pupae and adult [36]). Analysis of variance (ANOVA) was performed to evaluate the differences in BSF growth performance and physicochemical conditions among the three diets.

### Insect sampling, sterilization, and dissection.

BSF individuals fed on the three diets (FN, NRF, and NRV) were sampled at different developmental stages (Fig. S1): larvae were collected at the same instar as determined by head capsule width (75), pupae were collected based on the change in color from creamy-white to black, and both female and male adults were collected immediately after eclosion. Insects were sampled on different days based on their developmental stage (larvae, pupae, and adult) and unrelated to time. The surface was sterilized prior to insect dissection (76). Briefly, after a first wash with 0.1% sodium dodecyl sulfate in 50-mL tubes, BSF individuals were immersed in 1% sodium hypochlorite for 10 min, followed by three consecutive washes with 70% ethanol and five washes with sterilized distilled water. The gastrointestinal tracts of larvae and pupae were dissected under sterile conditions. For adults, due to morphogenetic events that affect the internal tissues of insects and the fragility of digestive tracts of the emerged individuals (36), we used the entire body after removal of the head, legs, and wings. For cultivation-independent analysis, a total of 81 samples (9 replicates per each diet at developmental stage) were separately collected and stored in 1.5-mL tubes containing 98% ethanol at −20°C.

### DNA extraction from diets and insects.

Metagenomic DNA was extracted from each sample using a DNeasy blood and tissue kit (Qiagen). The gut samples from larvae and pupae and adult bodies (without the head, legs, and wings) individually stored at −20°C were centrifuged for 5 min at 3,000 rpm. After ethanol removal, the tissues were hydrated by adding 1 mL of sterile physiological solution (0.9% NaCl). Samples were then centrifuged for 5 min at 3,000 rpm to remove the solution, and 180 μL of ATL buffer (DNeasy blood and tissue kit, Qiagen) was added. Tissues were homogenized in ATL buffer. To ensure digestion of the Gram-positive bacterial cell walls and the release of bacterial DNA, alternate incubations at −80°C and 70°C for 10 min each were performed. Then, 25 μL of lysozyme (20 mg/mL) was added to the homogenate and the samples were incubated at 37°C for 30 min. The final steps were performed following the manufacturer’s instructions. Sterile water was used as the control for the DNA extraction procedures to assess the presence of reagent contamination. The extracted DNA was used as the control in all the further molecular analyses. The quality of the extracted DNA was checked using a Nanodrop 1000 spectrophotometer (BioTek, PowerWave XS2). The feed substrates were also sampled (*n *= 3 per diet) in the absence of larvae in order to evaluate the initial bacterial composition. The substrates were crushed with liquid nitrogen, and the total DNA was extracted with the DNeasy PowerSoil Kit (Qiagen), following the manufacturer’s instructions.

### Fingerprinting and high-throughput sequencing of the bacterial communities.

The variation in the bacterial communities of insects exposed to different diets in each developmental stage was investigated using PCR fingerprinting (ARISA, automated ribosomal intergenic spacer analysis) and high-throughput sequencing (Illumina) of the ITS region and 16S rRNA gene, respectively. For ARISA-PCR fingerprinting, the ITS-F FAM (5′-GTC GTA ACA AGG TAG CCG TA-3′) and ITS-R (5′-GCC AAG GCA TCC ACC-3′) primer pairs were used (77), following the protocol described in Method S2; PCR mix (reagents without DNA) was used as control, and no amplification was detected by running the PCR product on 1.5% agarose gel. DNA extracts were further used to prepare Illumina libraries using the Illumina Nextera XT Sample Prep Kit and amplifying the V3 and V4 variable regions of the 16S rRNA gene with the primers 341f and 785r (78) as previously described in Mapelli et al. (79). DNA extracted from sterile water and PCR mix (reagent without DNA) was used as an additional control in the 16S rRNA gene amplicon PCR; no amplification was detected by running the PCR product on 1% agarose gel (Table S10). All tagged samples and controls were pooled and concentrated in a CentriVap DNA Concentrator (Labconco). The obtained libraries were sequenced using the MiSeq system with 2 × 300 base-pair read length in the Bioscience Core Lab at King Abdullah University of Science and Technology, Saudi Arabia. Sequence reads were deposited in the NCBI SRA database under SRA accession PRJNA421313. The raw data obtained from Illumina sequencing were analyzed using a combination of the Quantitative Insights into Microbial Ecology (QIIME) pipeline version 1.9 (80) and UPARSE version 8 (81), as described by Marasco et al. (67). Operational taxonomic units (OTUs) were formed based on 97% sequence identity cut-off values; representative sequences of each OTU were aligned in QIIME using UClust and searched against the SILVA 128 database for bacteria. OTUs present in blank and PCR mix controls were removed from all the samples within the data set (Table S10). For each sample, details on the read used in the subsequent analyses and Good’s coverage values are provided in Table S11.

Bray–Curtis (BC) dissimilarity distance matrices were obtained from the ARISA-ITS quantitative and the log-transformed-quantitative OTU matrices. Both BC matrices were used to perform PCoA (82) for each developmental stage (larvae, pupae, and adults [female and male]); no differences in the bacterial communities associated with female and male adults developed on the same diet were observed (Tables S4). Therefore, they were considered as belonging to the same group (i.e., the adult group) in our experimental design and further analyses. A multivariate generalized linear model (manyglm) (83) and multivariate linear model (manylm) (84) were used for the 16S rRNA gene and ITS data sets, respectively, considering diet as the fixed and orthogonal explanatory variable (three levels: FN, NRF, and NRV diets) for each developmental stage.

Using the 16S rRNA gene data set, the variance was partitioned using the *varpart* function within the Vegan package in R (85). Decay analysis was performed to evaluate the decrease in growth performance (i.e., weight) and bacterial community richness in larvae, pupae, and adults fed with the three diets according to the function of the gut bacterial community BC similarity. The cumulative proportion of bacterial community variation (R^2^), as explained by diet components (predictor variables), was calculated using the sequential test in addition to the corrected Akaike information criterion (86) within the distance-based multivariate analysis for a linear model (87). The components of beta diversity (similarity, replacement, and difference in richness) were calculated using the *beta.div.comp* function of the R package *adespatial* v0.3-8 (88). Alpha diversity indices were calculated using the Paleontological Statistics Software Package for education and data analysis (89). ANOVA and Tukey’s multiple comparison tests were performed to test differences in alpha diversity indices among the different diets across the three developmental stages using GraphPad software. The relative abundance and distribution of the OTUs were visualized with ternary plots using the *ggtern* software package in R (90). Nonparametric Kruskal–Wallis (false discovery rate, *p-*correction) and Dunn’s multiple comparisons tests were used to detect the differences in the taxonomic groups (class level) in the three diets across the developmental stages using GraphPad. The correlation between the taxonomic composition of the bacterial communities (class level) and diet components was determined using the combination of three methods (Pearson, Kendall, and Spearman) in R. Bacterial communities associated with the three feed substrates (i.e., FN, NRF, and NRV) were also analyzed to clarify the influence of feed-derived biotic factors in shaping bacterial communities associated with BSF.

### Co-occurrence network analysis.

To explore the significant relationships among the OTUs, a non-random structure of co-occurrence network was constructed for each bacterial community inhabiting the digestive tract of BSF fed with the three diets (FN, NRF, and NRV) (65) by considering (i) each developmental stage separately (larvae, pupae, and adults) and (ii) the overall life span of the insect (merging the three developmental stages) to detect conserved interactions induced by diet type (91). Although the analysis cannot distinguish between true ecological interactions and other nonrandom processes (for example, cross-feeding versus niche overlap), the correlation network can help to visualize the potential relationships between taxa within microbial communities (92, 93), especially in those cases in which inclusive functional culture-based studies result technically and logistically prohibitive (e.g., among others, uncultivability of majority of microbes and difficulties in obtain full-germfree individuals) (94). To analyze potential interactions within each pair of bacterial OTUs, we used the CoNet routine for Cystoscope following the methods described in Mapelli et al. (79). The topological indices of the networks were calculated using the network analyzer in Cytoscape (95) and the Cytoscape plug-in Centiscape 2.2 (96). For the overall life co-occurrence networks, visualization was performed using Gephi (97); statistical differences between the degrees of connection in the co-occurrence networks for the different diets were analyzed via ANOVA using R prior to performing the normality and homoscedasticity tests (98, 99). Nodes with a degree of connection > 75% were identified as network hubs, and those with the highest level of degree of connection and betweenness centrality were considered keystone species (44). Network stability was assessed as network robustness calculated with the R package NetSwan (100) using the function “*swan_combinatory ()*” by calculating the loss of connectivity among OTUs once nodes are removed. We stepwise removed the nodes from the network and recorded the loss of connections, measured as the decrease of the overall betweenness centrality and as decreased betweenness centrality recalculated in a cascading scenario after removal of each node (101, 102).

### Statistics and reproducibility.

Sequence data were processed in QIIME 1.9 and analyzed in PRIMER, GraphPad, and R. Detailed information on the number of replicates, statistics, and comparisons applied are provided in each Method section.

### Data availability.

The data set supporting the conclusions of this article are available in the NCBI SRA database, BioProject PRJNA421313, and in the Science Data Bank at http://www.doi.org/10.11922/sciencedb.01282.

### Code availability.

The R script used in this manuscript has been deposited in the Github community repository and is available at https://github.com/MarcoFusi1980/estabilization-of-the-bacterial-interactome-identifies-nutrient-restriction-induced-dysbiosis-in-in.git.
