# Supplementary material for: Destabilization of the Bacterial Interactome Identifies Nutrient Restriction-Induced Dysbiosis in Insect Guts
Source: Microbiol Spectr. 2022 Jan 5;10(1):e01580-21. doi: 10.1128/spectrum.01580-21 (PMC8729773; doi:10.1128/spectrum.01580-21)
Supplement: SUPPLEMENTAL FILE 1 — Supplemental material. Download SPECTRUM01580-21_Supp_1_seq9.pdf, PDF file, 1.3 MB [file spectrum01580-21_supp_1_seq9.pdf]

Supplementary Material of

Destabilization of the bacterial interactome identifies nutrient restriction-induced dysbiosis in insect guts

Ramona Marasco[1*], Marco Fusi[1*‡], Matteo Callegari[1*], Costanza Jucker[2], Francesca Mapelli[2], Sara Borin[2], Sara Savoldelli[2], Daniele Daffonchio[1#] and Elena Crotti[2#]

[1]Biological and Environmental Sciences and Engineering Division (BESE), Red Sea Research Center (RSRC), King Abdullah University of Science and Technology (KAUST), Thuwal 23955-6900, Kingdom of Saudi Arabia

[2]Department of Food, Environmental and Nutritional Sciences (DeFENS), University of Milan, Milan, Italy

[*]These authors contributed equally to this work

[#]Correspondence to Elena Crotti, elena.crotti@unimi.it and Daniele Daffonchio, daniele.daffonchio@kaust.edu.sa

[‡]Present Address: School of Applied Sciences, Edinburgh Napier University, Edinburgh, UK

Supplementary Methods

Supplementary Method S1. Measurement of physicochemical conditions of BFS gut comportments. Oxygen partial pressure (pO_2), pH and redox potential in the different compartments of the 4th instar BSF larvae gut (crop, foregut, midgut and hindgut) were measured using microsensors (Unisense, Aarhus, Denmark) on individuals fed with the three diets (FN, NRF and NRV). Oxygen microsensor (OX-50) had tip diameter of 50 µm. After an overnight polarization it was calibrated in water saturated with air in the CAL 300 calibration chamber (Unisense, Aarhus, Denmark), as well as in an anoxic solution of 0.1 M sodium dithionite. The pH microelectrode (PH-50) had a tip diameter of 50 µm and a sensitive tip length of 200 to 300 µm; it was calibrated using standard solutions at pH 4.0, 7.0 and 10.0. The redox microelectrode (RD-50) had a tip diameter of 50 µm; it was calibrated using saturated quinhydrone solutions at pH 4.0 and 7.0. In both cases, the electrode potentials were measured against Ag-AgCl reference electrodes using a high-impedance voltmeter (Ri > 1014 Ω). The current was measured with a Unisense microsensor multimeter and recorded using SensorTracePRO software (Unisense, Aarhus, Denmark). Before measurements, a freshly dissected gut was placed on the layer of 2% (Low Melting Point) agarose prepared with Ringer's solution (7.2 g/L NaCl; 0.37 g/L KCl; 0.17 g/L $CaCl_2$, pH 7.3–7.4) and immediately covered with a second layer of 0.5% agarose prepared with Ringer's solution. Microsensors were positioned using a motorized micromanipulator (Unisense, Aarhus, Denmark). All measurements were carried out at room temperature.

For each physicochemical parameter (pO_2, pH and redox potential) and alimentary regime (FN, NRF and NRV diets), seven larvae were analyzed. Permutational analysis of variance (PERMANOVA) was conducted to test differences of the physicochemical parameters among the gut traits (categorical explanatory variable fixed and orthogonal, four levels: Crop, Foregut, Midgut, Hindgut) and the different diets (categorical explanatory variable fixed and orthogonal, three levels: FN, NRF and NRV diets) by using PRIMER v.6.1, PERMANOVA+ for PRIMER routines [1–3].

Supplementary Method S2. ARISA fingerprinting analysis of bacterial communities Reaction mixture contained 1.50 U Taq DNA polymerase, 0.20 mM dNTP mixture, 0.30 µM of each primer, 1.5 mM $MgCl_2$ and 1 µl of DNA. The thermal protocol was the following: an initial denaturation step at 94°C for 3 minutes, followed by 30 cycles with a denaturation step at 94°C for 45 seconds, an annealing step at 55°C for 1 minute, an extension step at 72°C for 2 minutes and a final extension at 72°C for 7 minutes. PCR products were initially visualized in 1.5% agarose gel. ABI3730XL genetic analyser was used to separate ARISA fragments by applying the internal standard 1200-LIZ (Macrogen, Korea). The output peak matrix was transferred to Microsoft Excel for the following analysis. Peaks showing a height value <50 fluorescence units were removed from the output peak matrix before statistical analyses. Each polymorphic ARISA peak was defined as a different operational taxonomic unit (OTU). To account for variability in size associated with standards, ARISA fingerprints were binned ±1 bp from 150 to 300 bp, ±3 bp from 300 to 500 bp and ±10 bp >500 bp.

Supplementary Results

Supplementary Result S1. We detected a correlation between the relative abundance of the main bacterial classes and diet components and BSF growth performance (Table 2). For example, the relative abundance of *Bacilli* in larvae (dominating normobiotic microbiome) negatively correlated with moisture and positively correlated with other diet components (carbohydrates, proteins, lipids, fiber, and others) and larval weight. On the other hand, the relative abundance of *Gammaproteobacteria* and *Clostridia* (mainly detected in dysbiotic microbiomes) had an opposite trend. In pupae, while *Clostridia* had similar patterns of correlation with diet components, *Gammaproteobacteria*—dominant in FN-fed individuals—showed a negative correlation with moisture and a positive correlation with all other diet components and pupae weight. Furthermore, in adults, *Bacilli*, indicators of a normobiotic condition, had a positive correlation with host fitness (weight) and all components of FN diet, whereas *Clostridia* and *Gammaproteobacteria* had negative correlations.

Supplementary Tables

Supplementary Table S1. Chemical composition of rearing substrates: FN, full nutrient; NRF, nutrient restriction fruit; NRV, nutrient restriction vegetable. Values in the brackets indicate reduction (−) or increase (±) of nutrients' fresh weight expressed as percentage respect to FN as standard (100%).

**Fresh weight (g/100g)**	**FN[a]**	**NRF[b]**	**NRV[b]**
Moisture	55.1	86.6 (+57%)	92.3 (+68%)
Protein	10.4	0.4 (−96%)	2.0 (−81%)
Lipid	3.1	0.1 (−97%)	0.2 (−94%)
Carbohydrate	23.2	8.9 (−62%)	2.4 (−90%)
Fiber	5.6	2.7 (−52%)	2.3 (−59%)
Others	2.6	1.3 (−50%)	0.8 (−69%)
Carbohydrate/Protein	0.54	22.25	1.2

[a]Data were obtained for each ingredient of the diet by the provider and expressed on fresh weight.
[b]Data were taken from [4] and expressed on fresh weight.

Supplementary Table S2. Length of pupae and adult individuals. Lower case letters indicate the results of Tukey's multiple comparison tests. FN, full nutrient; NRF, nutrient restriction fruit; NRV, nutrient restriction vegetable.

**Length (mm)**	**FN**	**NRF**	**NRV**
Pupae	19.02 ± 1.47 (a)	15.48 ± 1.31 (b)	15.98 ± 1.87 (b)
Adult	17.34 ± 1.15 (a)	14.20 ± 1.14 (b)	14.80 ± 1.15 (b)

Supplementary Table S3. Correlation among the different components of the three diets (FN, NRF and NRV) and the weight of individuals at each developmental stage. Pearson, Kendall and Spearman correlation, $p<0.05$.

Diet component	Developmental stage		
	Larvae (n=18)	Pupae (n=180)	Adult (n=360)
Moisture	−0.92; *p*<0.001	−0.80; *p*<0.001	−0.84; *p*<0.001
Protein	0.93; *p*<0.001	0.80; *p*<0.001	0.86; *p*<0.001
Lipid	0.94; *p*<0.001	0.81; *p*<0.001	0.86; *p*<0.001
Carbohydrate	0.88; *p*<0.001	0.76; *p*<0.001	0.79; *p*<0.001
Fiber	0.93; *p*<0.001	0.80; *p*<0.001	0.85; *p*<0.001
Others	0.89; *p*<0.001	0.77; *p*<0.001	0.81; *p*<0.001

Supplementary Table S4. Difference among bacterial community associated with female and male are also assessed for each diet to ensure that all adult* can be analyzed as a unique developmental stage. Results of pairwise comparison (t-test) are reported for (**A**) 16S rRNA gene and (**B**) 16S-23S rRNA internal transcribed spacers (ITS) adult' datasets, respectively. Multivariate GLM analysis applied on the (**C**) 16S rRNA gene and (**D**) 16S-23S rRNA internal transcribed spacers (ITS) datasets. FN, full nutrient; NRF, nutrient restriction fruit; NRV, nutrient restriction vegetable.

(A) Adult*	Diet comparison	T	*p*(MC)
FN	Female vs Male	1.61	0.052
NRF	Female vs Male	1.52	0.054
NRV	Female vs Male	0.71	0.774

(B) Adult*	Diet comparison	T	*p*(MC)
FN	Female vs Male	1.72	0.057
NRF	Female vs Male	1.16	0.296
NRV	Female vs Male	0.81	0.502

(C) Stage	Res. Df	Df.diff	Dev.	*p*	Diet comparison	Res. Df	Df.diff	Dev.	*p*
					FN vs NRF	16	1	846	0.001
Larvae	24	2	4139	0.001	FN vs NRV	16	1	845	0.001
					NRF vs NRV	16	1	2489	0.001
					FN vs NRF	16	1	894	0.001
Pupae	21	2	2238	0.001	FN vs NRV	13	1	1178	0.001
					NRF vs NRV	13	1	1118	0.001
					FN vs NRF	16	1	647	0.004
Adult*	24	2	1335	0.001	FN vs NRV	16	1	609	0.002
					NRF vs NRV	16	1	625	0.004

(D) Stage	Res. Df	Df.diff	Dev.	*p*	Diet comparison	Res. Df	Df.diff	Dev.	*P*
					FN vs NRF	10	1	1898	0.002
Larvae	15	2	1571	0.002	FN vs NRV	10	1	1898	0.003
					NRF vs NRV	10	1	1610	0.003
					FN vs NRF	10	1	2396	0.002
Pupae	15	2	1197	0.002	FN vs NRV	10	1	1226	0.002
					NRF vs NRV	10	1	872	0.006
					FN vs NRF	10	1	1147	0.002
Adult*	15	2	707.5	0.002	FN vs NRV	10	1	738	0.006
					NRF vs NRV	10	1	507	0.038

Supplementary Table S5. Variation partitioning analysis of bacterial community explained by diets (FN, full nutrient; NRF, nutrient restriction fruit; NRV, nutrient restriction vegetable) at each developmental stage (larvae, pupae and adult) was assessed using *vegan* package in R [5].

Developmental stage	R^2 adjusted
Larvae	0.3603727
Pupae	0.3480033
Adult	0.1364717

Supplementary Table S6. Results of DistLM sequential tests of diet components (Supplementary Table S1) at each developmental stage (larvae, pupae and adult). Cumulative proportion of variation (R^2) in bacterial community structure explained by fitting variables (i.e., diet components) within sets sequentially using forward selection, and conditional tests using 999 permutations of residuals. Diet components included in the sequential test are reported, along with AICc, Pseudo-F, *p*-value and cumulative R^2.

Developmental stage	Diet component	AICc	Pseudo-F	*p*	Cumulative R^2
Larvae	Carbohydrates	195.4	15.227	0.001	0.378
	Others	172.6	37.288	0.001	0.756
Pupae	Proteins	178.4	10.131	0.001	0.315
	Moisture	165.6	18.91	0.001	0.639
Adult	Carbohydrates	210.4	4.855	0.001	0.162
	Moisture	208.7	4.011	0.001	0.282

Supplementary Table S7. Diversity indices describing the bacterial community associated to BSF subjected to different diet regimes along the development stages (larvae, pupae and adult). *Richness* indicates the total number of OTUs for each sample; *Evenness* is represented by Pielou's index and quantifies how equal the community is numerically. All the values are reported as average ± standard deviation; number of replicates indicated as (n). Lower case letters indicate the results of multiple comparison tests for each developmental stage. FN, full nutrient; NRF, nutrient restriction fruit; NRV, nutrient restriction vegetable.

Developmental stage	Diet (n)	Richness	Evenness
Larvae	FN (9)	46 ± 10 (a)	0.04 ± 0.02 (a)
	NRF (9)	28 ± 8 (b)	0.09 ± 0.05 (b)
	NRV (9)	122 ± 21 (c)	0.14 ± 0.05 (c)
Pupae	FN (9)	48 ± 11 (a)	0.06 ± 0.04 (a)
	NRF (9)	29 ± 5 (b)	0.07 ± 0.04 (a)
	NRV (6)	73 ± 20 (c)	0.15 ± 0.08 (b)
Adult	FN (9)	43 ± 16 (a)	0.09 ± 0.05 (a)
	NRF (9)	53 ± 20 (a)	0.18 ± 0.12 (a)
	NRV (9)	38 ± 12 (a)	0.09 ± 0.05 (a)

Supplementary Table S8. Within beta-diversity components (species replacement and richness difference) expressed as average ± standard deviations of the pairwise similarities of each diet (FN, full nutrient; NRF, nutrient restriction fruit; NRV, nutrient restriction vegetable) along the developmental stages (larvae, pupae and adult). Ratio among replacement and richness difference processes is also reported. Star (*) indicate the case in which replacement not dominate on richness difference. Relative contribution of both components is expressed in percentage and reported in the brackets. Lower case letters indicate the results of the Tukey's multiple comparison test for replacement (upper panels) and richness difference (lower panels) at each developmental stage.

Beta-diversity component	Stage	FN	NRF	NRV
Richness difference	Larvae	0.201 ± 0.127 (39%) a	0.246 ± 0.15 (45%) a	0.160 ± 0.101 (42%) a
	Pupae	0.191 ± 0.138 (37%) a	0.141 ± 0.087 (26%) b	0.250 ± 0.131 (55%) b
	Adult	0.304 ± 0.186 (44%) b	0.226 ± 0.179 (30%) c	0.271 ± 0.215 (44%) a
Replacement	Larvae	0.310 ± 0.116 (61%) ab	0.304 ± 0.148 (55%) ab	0.218 ± 0.097 (58%) a
	Pupae	0.329 ± 0.098 (63%) a	0.398 ± 0.106 (74%) a	0.203 ± 0.096 (45%) a
	Adult	0.392 ± 0.172 (56%) b	0.524 ± 0.195 (70%) b	0.350 ± 0.196 (56%) a
Replacement/ Richness difference	Larvae	1.5	1.2	1.4
	Pupae	1.7	2.8	0.8*
	Adult	1.3	2.3	1.3

Supplementary Table S9. Co-occurrence network topology indices reported for larvae (L), pupae (P) and adults (A) fed on FN (full nutrient), NRF (nutrient restriction fruit) and NRV (nutrient restriction vegetable) diets. Star (*) indicate the indices expressed as percentage of increment (+) or decrement (–) respect to the FN.

Topology	L-FN	L-NRF	L-NRV	P-FN	P-NRF	P-NRV	A-FN	A-NRF	A-NRV
Nodes	65	67	147	68	35	111	71	138	88
Interactions	577	837	1700	549	143	1375	1033	1297	899
Positive	357	407	1079	358	89	956	666	1026	628
Negative	220	430	621	191	54	419	367	271	270
Mean of degree	18±9 a	25±12 b	23±19 ab	16±7 a	8±4 b	25±12 c	30±9 a	19±8 b	19.5±8 b
Cluster coefficient*		+16.71	+0.08		+32.52	+30.26		+8.65	+2.39
Centralization*		–15.83	−45.41		−49.95	−29.79		−36.19	−27.01
Av. path length*		−9.52	+39.16		+22.07	+4.03		+45.44	+20.55
Av. neighbours*		+40.73	+30.28		−49.75	+74.52		−35.40	−29.86
Density*		+0.42	−6.84		−0.97	+6.30		−66.99	−43.57
Heterogeneity*		+39.39	+72.80		+13.94	+24.04		+44.03	+32.73

Supplementary Table S10. Negative control samples included to account for contaminations during the experimental workflow. Controls were performed starting from the blank of DNA extraction done with sterile water as sample; these samples were further used throughout the PCR amplification and sequencing; control of PCR mix (al reagent without DNA) are also included in the sequencing reads analysis. OTUs detected within negative control samples were removed from all samples; number of OTUs and corresponding reads removed from all samples are reported in the table.

Analysis	
PCR amplification from blank DNA	Band not visible in the gel 1% agarose
PCR amplification from PCR mix	Band not visible in the gel 1% agarose
Index PCR of bacterial amplicon from blank DNA	Band not visible in the gel 1.2% agarose
Index PCR of bacterial amplicon from PCR mix	Band not visible in the gel 1.2% agarose
Bacterial OTUs in blank DNA	22 OTUs, total 9,715 reads removed from the dataset
Bacterial OTUs in PCR mix	6 OTUs, total 3,210 reads removed from the dataset

Supplementary Table S11. Sequence analysis and bacterial OTUs selection. For each sample, number of reads for the different categories are reported, along with the number of OTUs corresponding to that category. Good's coverage values are also listed. FN, full nutrient; NRF, nutrient restriction fruit; NRV, nutrient restriction vegetable

ID	Stage	Diet	Reads used	N. OTUs	Good's coverage value
LFN1	Larvae	FN	155594	48	99.99
LFN2	Larvae	FN	115026	63	99.99
LFN3	Larvae	FN	151889	48	99.99
LFN4	Larvae	FN	151139	44	99.99
LFN5	Larvae	FN	135962	49	99.99
LFN6	Larvae	FN	148697	39	99.99
LFN7	Larvae	FN	132790	37	99.99
LFN8	Larvae	FN	16345	31	99.94
LFN9	Larvae	FN	15364	29	99.95
LNRF1	Larvae	NRF	186775	43	100.00
LNRF2	Larvae	NRF	184974	42	99.99
LNRF3	Larvae	NRF	147454	42	99.99
LNRF4	Larvae	NRF	79575	30	99.99
LNRF5	Larvae	NRF	178473	45	99.99
LNRF6	Larvae	NRF	169555	62	99.98
LNRF7	Larvae	NRF	95326	34	99.99
LNRF8	Larvae	NRF	16952	23	99.98
LNRF9	Larvae	NRF	23812	26	99.99
LNRV1	Larvae	NRV	426781	155	100.00
LNRV2	Larvae	NRV	116001	107	99.98
LNRV3	Larvae	NRV	116103	99	99.99
LNRV4	Larvae	NRV	491208	141	100.00
LNRV5	Larvae	NRV	280834	113	99.99
LNRV6	Larvae	NRV	128575	104	99.99
LNRV7	Larvae	NRV	100550	111	99.98
LNRV8	Larvae	NRV	326158	155	100.00
LNRV9	Larvae	NRV	33435	127	99.96
PFN1	Pupae	FN	163761	55	99.99
PFN2	Pupae	FN	121469	56	99.99
PFN3	Pupae	FN	132035	54	99.99
PFN4	Pupae	FN	213587	43	100.00
PFN5	Pupae	FN	142119	59	99.99
PFN6	Pupae	FN	156635	56	99.99

PFN7	Pupae	FN	6057	29	99.87
PFN8	Pupae	FN	8901	36	99.83
PFN9	Pupae	FN	12902	42	99.89
PNRF1	Pupae	NRF	80545	30	99.99
PNRF2	Pupae	NRF	63933	32	99.99
PNRF3	Pupae	NRF	106038	37	99.99
PNRF4	Pupae	NRF	66967	34	99.98
PNRF5	Pupae	NRF	71926	28	99.99
PNRF6	Pupae	NRF	67352	30	99.98
PNRF7	Pupae	NRF	10436	23	99.91
PNRF8	Pupae	NRF	21042	24	99.99
PNRF9	Pupae	NRF	9315	22	99.91
PNRV1	Pupae	NRV	31986	105	99.95
PNRV2	Pupae	NRV	57426	77	99.98
PNRV3	Pupae	NRV	80806	83	99.99
PNRV4	Pupae	NRV	7202	48	99.90
PNRV5	Pupae	NRV	2921	71	99.35
PNRV6	Pupae	NRV	6334	56	99.81
AFN1	Adult	FN	131059	57	99.99
AFN2	Adult	FN	2644	57	99.17
AFN3	Adult	FN	12793	36	99.94
AFN4	Adult	FN	5828	18	99.97
AFN5	Adult	FN	127263	70	99.99
AFN6	Adult	FN	141429	50	99.99
AFN7	Adult	FN	166987	44	99.99
AFN8	Adult	FN	26840	23	99.97
AFN9	Adult	FN	6076	38	99.79
ANRF1	Adult	NRF	120532	45	100.00
ANRF2	Adult	NRF	2306	37	99.65
ANRF3	Adult	NRF	22820	43	99.97
ANRF4	Adult	NRF	2076	40	99.57
ANRF5	Adult	NRF	83265	92	99.98
ANRF6	Adult	NRF	23395	75	99.98
ANRF7	Adult	NRF	26704	41	99.96
ANRF8	Adult	NRF	74232	73	99.98
ANRF9	Adult	NRF	4954	38	99.92
ANRV1	Adult	NRV	147059	56	99.99
ANRV2	Adult	NRV	75181	47	99.99
ANRV3	Adult	NRV	82403	31	99.99
ANRV4	Adult	NRV	3634	14	99.83
ANRV5	Adult	NRV	117875	28	99.99
ANRV6	Adult	NRV	35588	44	99.96
ANRV7	Adult	NRV	102878	41	99.99
ANRV8	Adult	NRV	90061	43	100.00
ANRV9	Adult	NRV	13275	41	99.95
FN1	Diet	FN	58102	580	99.95
FN2	Diet	FN	6469	387	98.76
FN3	Diet	FN	20996	466	99.68
NRF1	Diet	NRF	8710	43	99.78
NRF2	Diet	NRF	6710	38	99.87
NRF3	Diet	NRF	26737	59	99.94
NRV1	Diet	NRV	27623	291	99.92
NRV2	Diet	NRV	29151	272	99.92
NRV3	Diet	NRV	33014	313	99.94

Supplementary Figures

Supplementary Figure S1. Representative images of BFS larvae, pupae (black color) and adults fed on full nutrient (FN), nutrient restriction fruit (NRF) and nutrient restriction vegetable (NRV). Bar scale indicate 10 mm.

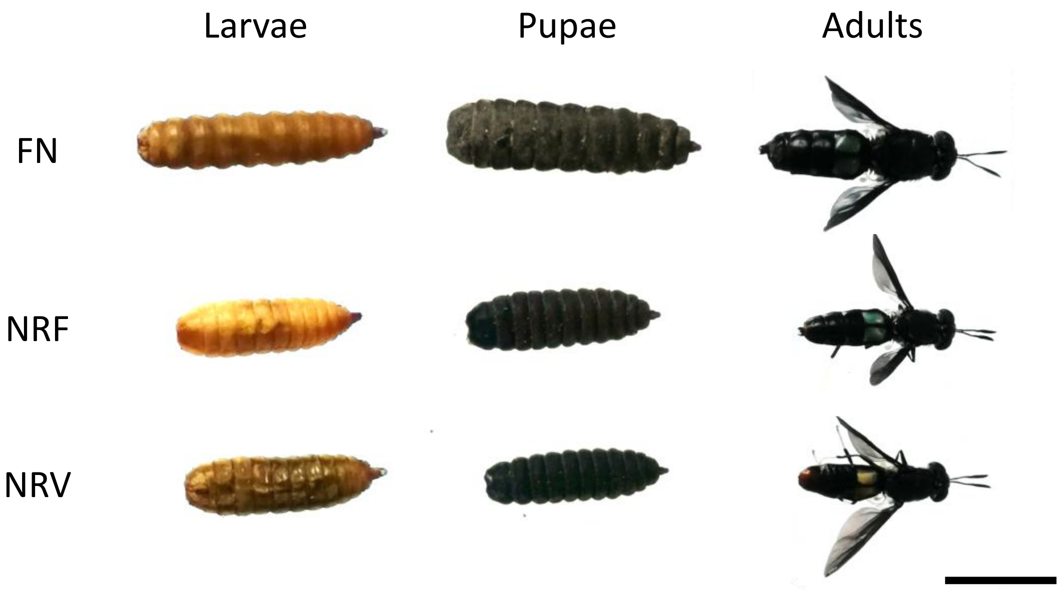

Supplementary Figure S2. PCoA-analysis based on ARISA Bray-Curtis dissimilarities matrices of gut' bacterial communities associated with (**A**) larvae, (**B**) pupae and (**C**) adults fed on FN (full nutrient, black), NRF (nutrient restriction fruit, orange), and NRV (nutrient restriction vegetable, green).

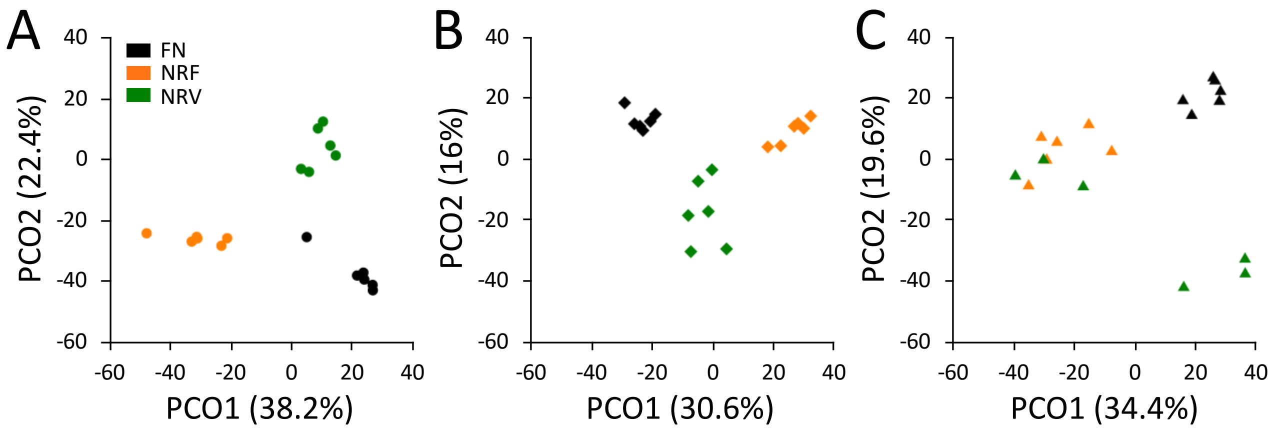

Supplementary Figure S3. Relationship between bacterial communities' similarities (Bray-Curtis) and the weight decay of (**A**) larvae, (**B**) pupae, and (**C**) adults fed on full nutrient and nutrient restriction diets. R-squared values of regression line and statistical significance of correlation among the two variables (*p*<0.05) are shown in the graph.

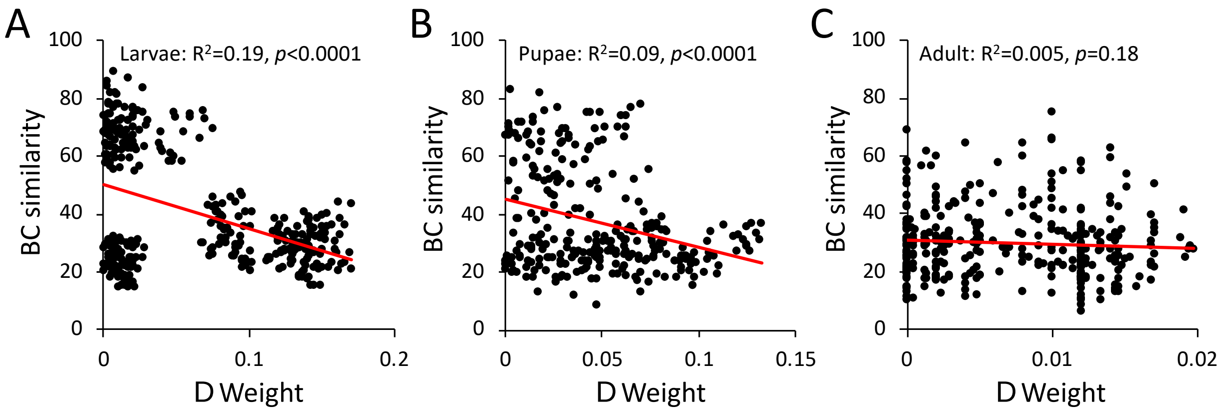

Supplementary Figure S4. Relationship between bacterial communities' similarities (Bray-Curtis) and the richness decay of (**A**) larvae, (**B**) pupae, and (**C**) adults fed on the different diets (full nutrient, nutrient restriction fruit and nutrient restriction vegetable). R-squared values of regression line and statistical significance of correlation among the two variables (*p*<0.05) are shown in the graph.

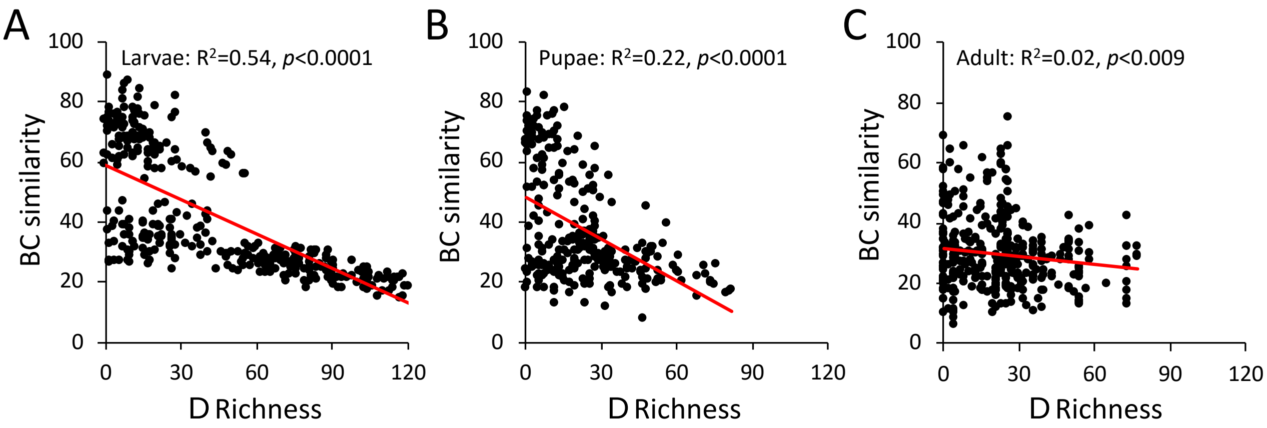

Supplementary Figure S5. Relationship between within beta-diversity (*i.e.*, dispersion) and the richness (number of OTUs) of gut bacterial communities associated with (**A**) larvae, (**B**) pupae, and (**C**) adults fed on the different diets (full nutrient, nutrient restriction fruit and nutrient restriction vegetable). R-squared values of regression line and statistical significance of correlation among the two variables ($p<0.05$) are shown in the graph.

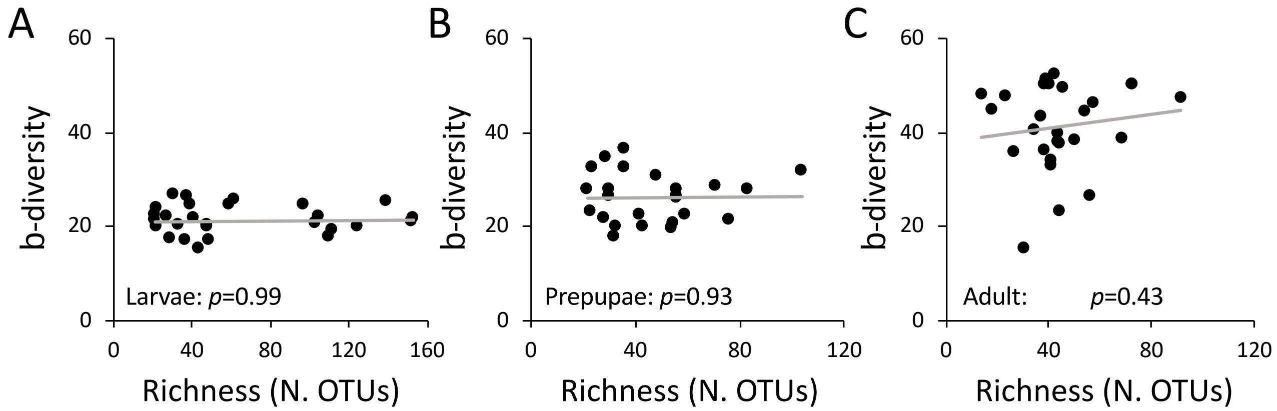

Supplementary Figure S6. (**A**) Heatmap of bacterial classes' relative abundance (%) in larvae, pupae and adults fed on FN and NR diets. (**B**) Relative abundance of the bacterial class across the three diets (n = 3 for each diet). Relative abundance is expressed as percentage of sequence frequency; FN, full nutrient (black); NRF, nutrient restriction fruit (orange); and NRV, nutrient restriction vegetable (green).

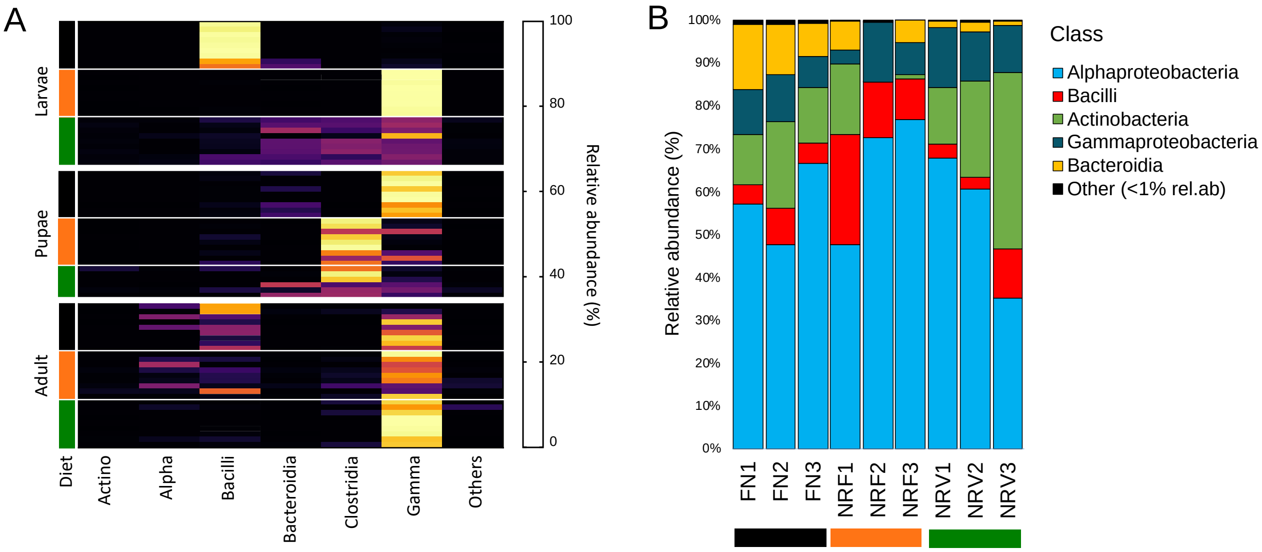

Supplementary Figure S7. Heatmaps showing the relative abundance of all the bacterial OTUs across the 78 samples analyzed; (**A**) OTUs with an overall relative abundance ≥ 0.02%; (**B**) OTUs with relative abundance < 0.02%. Percentages of relative abundance are reported as log-transformed values (range, from 0 to 2). Refer to the Supplementary Data S1 for the taxonomic affiliation of the OTUs.

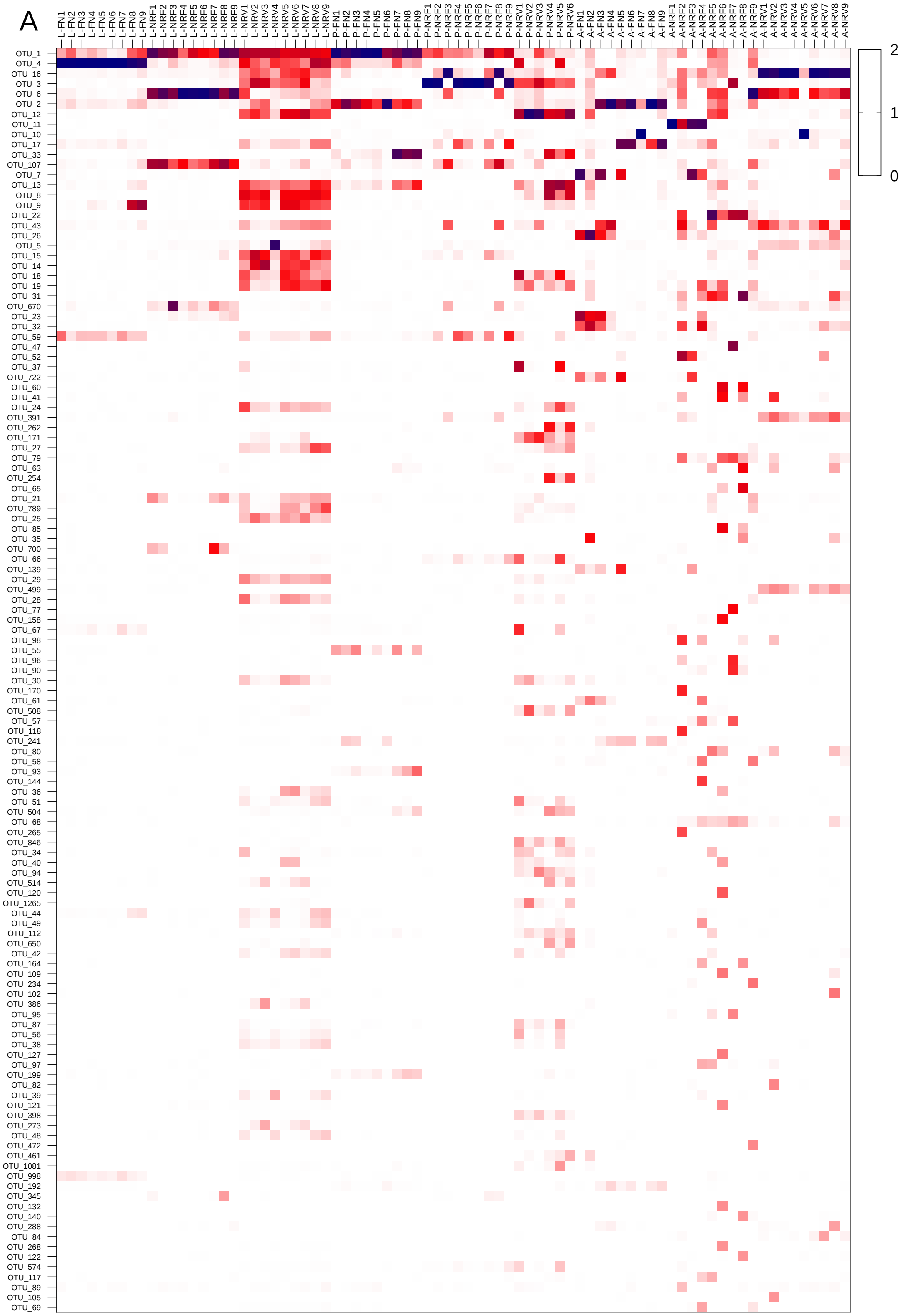

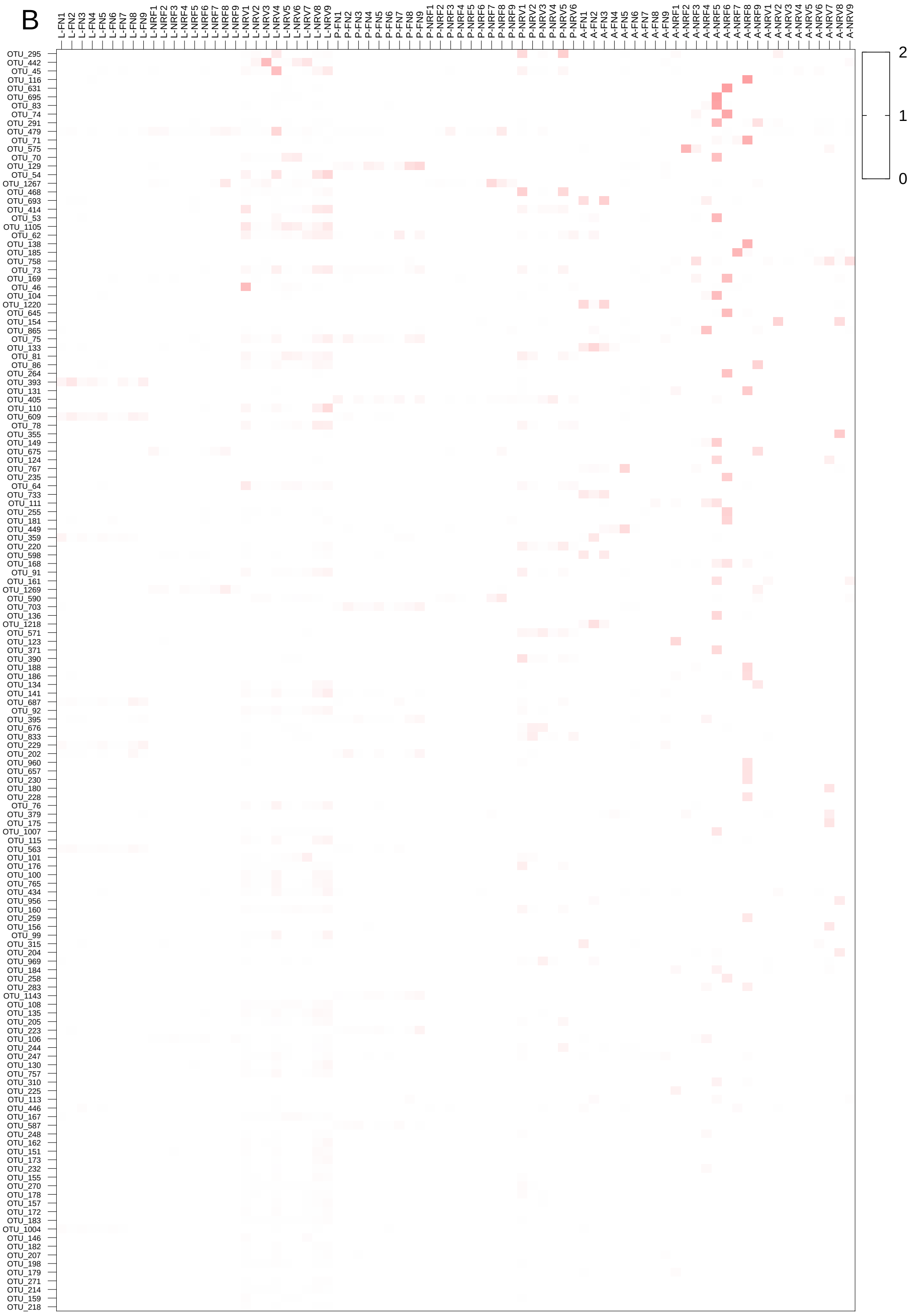

Supplementary Figure S8. Relative abundance of the main bacterial classes detected in BFS fed with FN and NR diets. Lower case letters indicate the results of the Dunn's multiple comparison test among diets at each developmental stage. FN, full nutrient (black); NRF, nutrient restriction fruit (orange); and NRV, nutrient restriction vegetable (green).

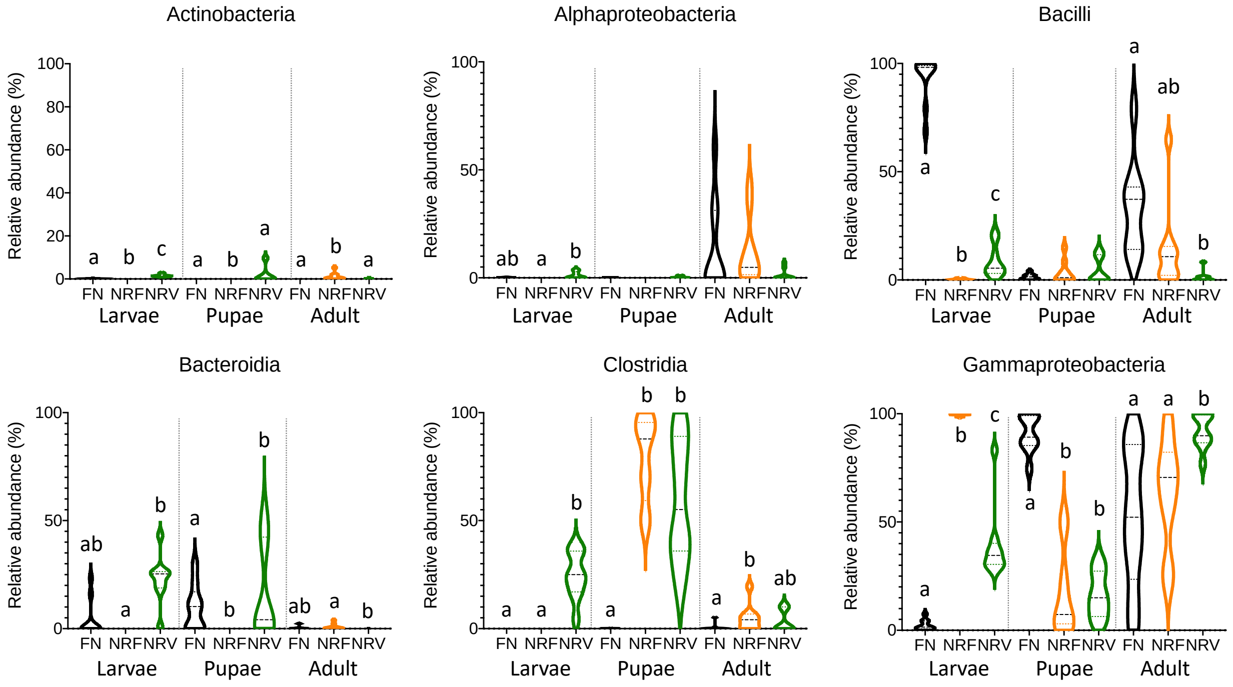

Supplementary Figure S9. Relative abundance of the main bacterial classes detected in BFS fed with FN and NR diets. Results are reported for the (**A**) total bacterial communities (total number of reads), (**B**) networks' nodes (n indicate the total number of network nodes) and (**C**) networks' hubs (n indicates the total number of network hubs).

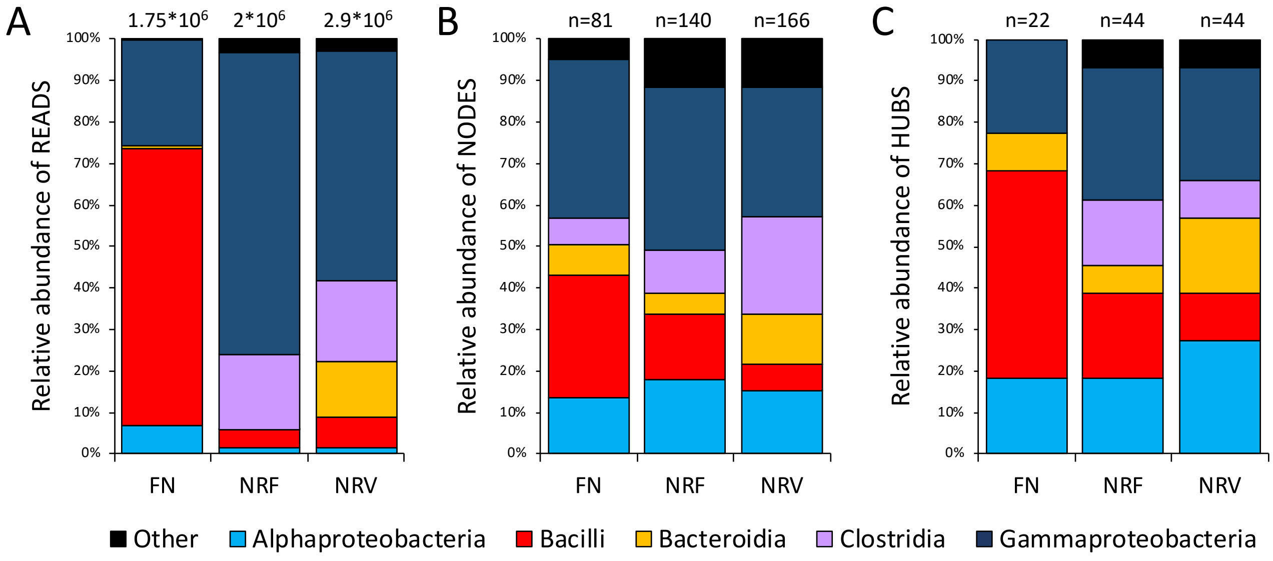

Supplementary Data

Supplementary Data S1. Relative abundance and taxonomy of bacterial OTUs in larvae, pupae, adult and diet samples. Refer to Marasco et al 2021_Supplementary Data S1.xlsx

Supplementary References

1. Ramette A. Multivariate analyses in microbial ecology. *FEMS Microbiol Ecol* . 2007; **62**: 142–160
2. Buttigieg PL, Ramette A. A guide to statistical analysis in microbial ecology: a community-focused, living review of multivariate data analyses. *FEMS Microbiol Ecol* 2014; **90**: 543–550.
3. Anderson MMJJ, Gorley RNRN, Clarke KRR. PERMANOVA + for PRIMER: Guide to software and statistical methods; PRIMER-E. *PRIMER-E Ltd*. 2008. Plymouth, UK.
4. Jucker C, Erba D, Leonardi MG, Lupi D, Savoldelli S. Assessment of vegetable and fruit substrates as potential rearing media for *Hermetia illucens* (Diptera: Stratiomyidae) larvae. *Environ Entomol* 2017; **46**: 1415–1423.
5. Oksanen AJ, Blanchet FG, Friendly M, Kindt R, Legendre P, Mcglinn D, et al. Package ‘vegan’ (Version 2.4-0). 2016.
